# What is the function of mitochondrial networks? A theoretical assessment of hypotheses and proposal for future research

**DOI:** 10.1002/bies.201400188

**Published:** 2015-04-03

**Authors:** Hanne Hoitzing, Iain G Johnston, Nick S Jones

**Affiliations:** Department of Mathematics, Imperial College LondonLondon, UK

**Keywords:** hypotheses, mathematical biology, mitochondrial dynamics, mitochondrial networks, non-linearities, ultrastructure

## Abstract

Mitochondria can change their shape from discrete isolated organelles to a large continuous reticulum. The cellular advantages underlying these fused networks are still incompletely understood. In this paper, we describe and compare hypotheses regarding the function of mitochondrial networks. We use mathematical and physical tools both to investigate existing hypotheses and to generate new ones, and we suggest experimental and modelling strategies. Among the novel insights we underline from this work are the possibilities that (i) selective mitophagy is not required for quality control because selective fusion is sufficient; (ii) increased connectivity may have non-linear effects on the diffusion rate of proteins; and (iii) fused networks can act to dampen biochemical fluctuations. We hope to convey to the reader that quantitative approaches can drive advances in the understanding of the physiological advantage of these morphological changes.

## Introduction

Mitochondria are highly dynamic organelles of central importance for ATP production in most eukaryotic cells. They are observed to undergo fusion and fission events continuously, leading to a diverse range of mitochondrial morphologies, from fragmented states to continuous networks. Mitochondrial dynamics and morphological structures have been the object of intense study in the past two decades: they are fundamental to the functionality of the cell, highly responsive to cellular state 1 and have been implicated in numerous diseases including Parkinson’s 2, diabetes 3, cancer 4 and Alzheimer’s 5, as well as being of central importance in various mitochondrial diseases 3,6,7. Despite this medical importance – and the increasing volume and detail of experimental results describing mitochondrial networks – many aspects of the function of mitochondrial fission and fusion remain unclear.

**Figure 1:**
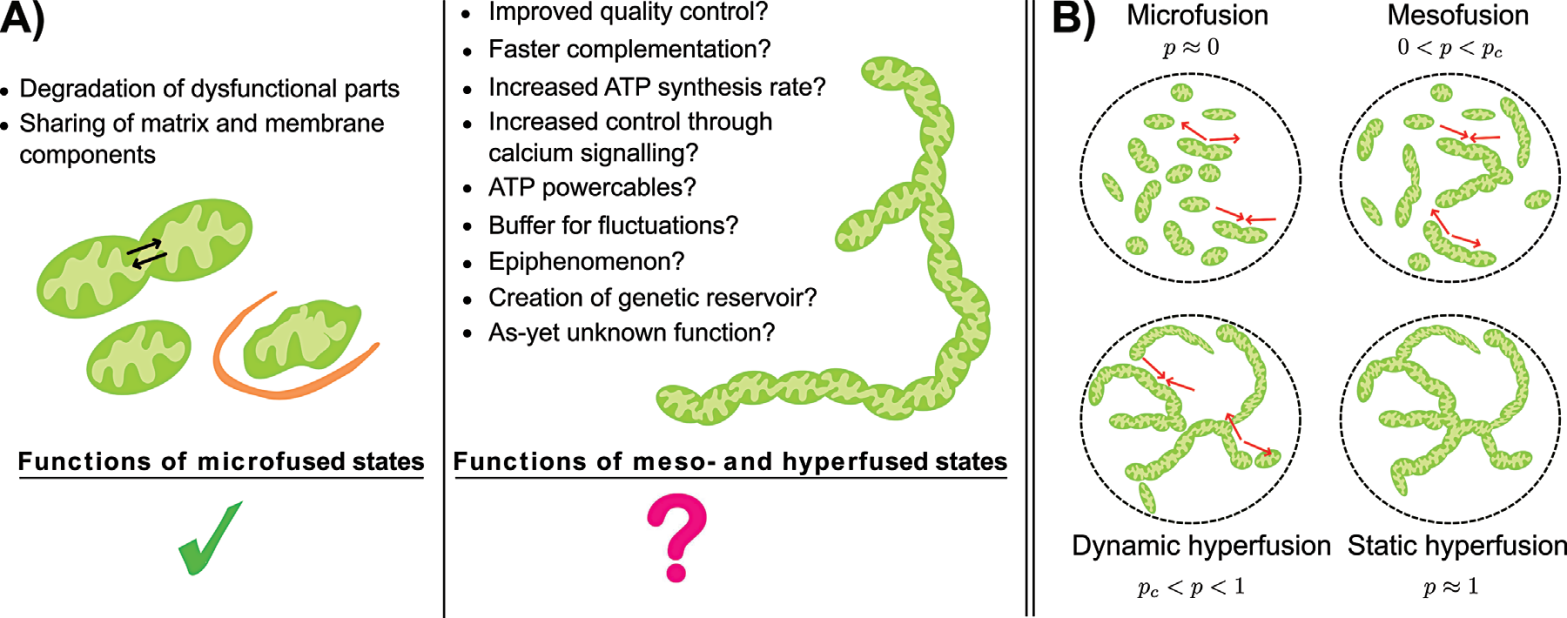
Potential causes and types of mitochondrial network formation. A: There are clear benefits of small-scale fusion events (microfusion) such as membrane and matrix protein complementation (and possibly mtDNA complementation) and selective degradation. However, to appreciate these advantages, one does not require the formation of large extended networks, the functions of which remains to be elucidated. In this paper we discuss the functions proposed in the right panel. B: The quantity *p*, defined as *λ*_fus_/(*λ*_fus_ + *λ*_fis_), roughly estimates the probability that any two neighbouring mitochondrial units are fused. Note that different rates of fusion and fission can lead to the same connectivity of the network, as long as the ratio *λ*_fus_/(*λ*_fus_ + *λ*_fis_) remains constant. In a static hyperfused state, virtually no fission events occur and therefore no quality control is possible. However, in a dynamic hyperfused state the fission rate is non-zero (*λ*_fis_ > 0) and quality control is present. The red arrows represent fusion or fission events and are absent in the static hyperfused state.

Various effects of fused mitochondrial states have been observed, including an increase in energy production 8–12, protection against apoptotic stresses 10,13–16, an increase in cell proliferation (17 and references therein), and regulation of various signalling pathways 18–21. Exactly how these effects are established through increased mitochondrial connectedness remains unclear. Here, we attempt to provide more clarity by exploring the mechanistic advantages of mitochondrial networks (i.e. large connected pieces of mitochondrial material) including increases in controllability, efficiency, robustness of mitochondria with respect to perturbations, and increases in oxidative capacity. To complement the mainly qualitative discussions that exist in the literature, we here use mathematical and physical arguments to probe the properties and bioenergetics of mitochondrial network formation, with the aim of gaining biological insights and suggesting research strategies. In the right-hand panel of Fig. 1A, several possible functions of mitochondrial networks are proposed. Some of them are novel; others have been suggested based on experimental results, and discussed qualitatively, largely in the absence of mathematical models to test them. We hope to show that quantitative approaches to the question of mitochondrial network formation can serve as a tool to critically analyse old hypotheses and motivate new ones.

We will not focus on the specific proteins involved in mitochondrial dynamics, but we occasionally refer to them and therefore provide a very brief overview. The three main proteins involved in the fusion of mammalian mitochondria are the GTPases Optic Atrophy 1 (OPA1) and the two mitofusins, MFN1 and MFN2. The central protein in mitochondrial division is the highly conserved Dynamin-Related Protein 1 (Drp1), which also belongs to the family of large GTPases. For a review on the proteins involved in mitochondrial dynamics, readers can refer to Ref. 22.

## Quantitative definitions of micro, meso- and hyperfusion

To assist in developing intuition describing our approach, we will assume here that there exists a smallest mitochondrial ‘unit’. These units may be fused (when they are part of a lumenally continuous filament) or fragmented. This assumption is a mathematical convenience and does not affect our later findings or represent our belief in a quantised set of mitochondrial sizes. We define a fission rate *λ*_fis_ and a fusion rate *λ*_fus_. These rates are assumed to be constants per mitochondrial unit (this automatically makes both fission and fusion rates larger in a long mitochondrial filament than in a small filament because the larger filament consists of more units, each with rates *λ*_fis_ and *λ*_fus_). The quantity 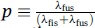 then broadly estimates the probability that a continuous link exists between any two neighbouring mitochondrial units (i.e. the probability that two neighbouring units are fused).

Percolation theory 23 suggests that as *p* increases, at some value of *p* a percolating path (a chain of fused mitochondria extending from one end of the cell to the other, in the limit of very large cells) will exist. This situation merits some special consideration in the context of several hypotheses, and so we refer to this percolating value as *p*_c_ (the exact value of *p*_c_ depends on the underlying network structure). Roughly, at and above this critical value, a mitochondrion at one extreme end of the cell is expected to be continuously connected to a mitochondrion at the other end of the cell. We note that simple percolation theory is of relatively limited use in describing fine detail of mitochondrial dynamics, which are dynamic, unlikely to lie on a lattice, and have connectivity properties dependent on membrane potential.

The different morphological states we introduce are fragmented (*p* = 0; no fusion), microfused (*p* ≈ 0; rare fusion), mesofused (0 < *p* < *p*_c_; fission dominated), dynamic hyperfused (*p*_c_ < *p* < 1; fusion dominated) and static hyperfused (*p* ≈ 1; rare fission). These states are illustrated in Fig. 1B. A high value of *p* means that the mitochondrial population is in a highly fused state, which can be the result of a high fusion rate, low fission rate, or both. The distinction between dynamic hyperfusion and static hyperfusion is important. For both hyperfused states a percolating continuous path exists (*p* > *p*_c_). The difference is that in dynamic hyperfused states, there can still be an appreciable rate of fission (e.g. if fusion proteins are overexpressed), whereas in static hyperfused states there are (almost) no fission events (e.g. if fission proteins are knocked out or heavily downregulated). Enforcing mitochondrial elongation leads to oxidative damage and decreased respiration 24, increased cell death 25, mitochondrial DNA (mtDNA) loss 26 and chromosomal instability 27. In many of these studies, the hyperfused state is induced by knockout of fission proteins, which creates a static hyperfused state. However, if physiological hyperfusion involves dynamic rather than static hyperfusion, fission events do occur and the aforementioned negative consequences may not be manifested.

We appreciate that our assumption of equal rates of fission and fusion for all mitochondrial units represents an oversimplification. In reality, for example, fusion rate depends on membrane potential 24,28,29 and fission and fusion events are not independent 24,30. We nonetheless believe that using a simplified approach can help make testable predictions and it provides a framework for more sophisticated models. We note that there is a disconnect between an idealised mathematical description of network structure and experimental measurements that are currently possible, although new approaches are narrowing this gap 31,32, but useful insights may still be gained by considering mathematical abstractions.

Physiological circumstances in which meso- and hyperfusion have been observed, as well as the ‘typical’ morphological state of mitochondria, are summarized in Tables S1 and S2: it is currently unclear whether meso- and hyperfusion are widespread cellular phenomena or restricted to certain cell types and situations.

## Hypothesised reasons for mitochondrial meso- and hyperfusion

In this section, we list existing hypotheses regarding the function of mitochondrial meso- and hyperfusion. Besides the hypotheses discussed here, additional hypotheses that could not be included due to space restrictions are mentioned in the supplementary information (SI) in sections S2.1–S2.4. These include the possibilities that meso- and hyperfusion (i) create a genetic reservoir 33; (ii) increase ATP synthesis through a range of mechanisms in addition to the one described in the main text; (iii) enable faster energy transmission along mitochondrial cables 34–37 (see also Fig. 3C); and (iv) have no function. All hypotheses (including the ones discussed in the SI) are summarized in Table1, which includes critiques of each hypothesis. In Table2 we provide, for each hypothesis discussed in the main text, experimental tests and suggestions for further modelling.

**Table 1:** An overview of hypotheses discussed in this paper including criticism

Hypothesis and references	Limitations/criticism
Increased selection bias in quality control^*^	The results of the model that is used depend on the rate of autophagy. If no autophagy is present, one needs to create additional assumptions to obtain the same results
Faster or more effective complementation 38–43	Matrix protein complementation through small fusion events is efficient 43, cells with low fusion levels show no major dysfunctions 44, and ongoing fission and fusion events can, according to one estimate, lead to better mixing after 2 hours than stress induced hyperfusion 41
Increases in ATP production caused by:	
(i) Changes in inner membrane shape (discussed in section S2.2.1) 45–50	(i) There is no obvious reason why cristae shape should be determined by overall state of mitochondrial organisation
(ii) Decreases in proton leak (discussed in section S2.2.2) 51	(ii) This may only be relevant in brown adipose tissue (because proton leak has a more important role in this tissue); it may not explain hyperfusion in other tissues
(iii) Decreases in mitophagy levels (discussed in section S2.2.3) 9,12	(iii) The cell may keep total mitochondrial mass (or mitochondrial volume) at the same level; an absence of mitophagy may have other undesirable cellular consequences
(iv) Non-linear response of ATP synthesis rate to membrane potential^*^	iv) The model discussed considers the role of Δ*ψ* in producing ATP, whereas actually the proton motive force drives ATP synthesis: extensions to the electrochemical potential are desirable
Improved bioenergetic control and energy production through Ca^2+^ signalling 52–55	Various studies suggest that calcium has no significant influence on rate of ATP synthesis in vivo 56. Other modes of calcium uptake besides the mitochondrial uniporter that are more rapid exist 57
Increased buffering against perturbations^*^	It might seem just as natural that in a fragmented state, the isolation of the different mitochondria is a form of robustness. Fluctuations in larger mitochondria will occur more frequently because of the larger surface area
Enables energy transmission (power cabling) along mitochondria (discussed in section S2.3) 35–37	This does not account for hyperfusion in tissues that are less dependent on oxygen and have lower ATP demand
Creating a genetic reservoir (discussed in section S2.1) 33^*^	It is not clear whether large-scale fusion is necessary to maintain a genetic reservoir; modulating biogenesis and mitophagy might be sufficient. Some mitochondria with harmful mtDNA mutations may not be able to fuse, and are likely to be degraded regardless of increased fusion rates. If mtDNA mutations are not harmful, increased fusion is not required per se to create new mutations
No function (discussed in section S2.4)	It seems coincidental that different kinds of stress lead to increases in fusion or decreases in fission activity with a hyperfused state as a result. Additionally, the main argument to support this hypothesis (many proteins involved in mitochondrial dynamics are involved in other processes) is also an argument for the importance of mitochondrial dynamics

Asterisks denote hypotheses that, to our knowledge, have not been previously proposed.

**Table 2:** Suggestions for future modelling and experiments for further analyses of the hypotheses

Hypothesis for forming mitochondrial networks	Further modelling	Experimental tests
Increased selection bias in quality control	The ordinary differential equation model we describe in section S1.1 is deterministic and neglects the pronounced stochastic influences likely to affect mitochondrial quality control. More powerful models could be constructed by including these stochastic influences and relaxing some of the simplifying assumptions of our model	One of the assumptions of our model is that only small mitochondrial fragments are degraded. The existence of a threshold size above which a mitochondrial filament is not degraded by mitophagy can be measured. Alternatively, construct two populations of cells, one wild-type and one with increased fusion rates. The autophagy rate parameter should be the same in both populations. Measure the average Δ*ψ* in cells from both populations. Our model predicts that the distribution of averages is more centered towards low values of Δ*ψ* in the wild-type population
Faster or more effective complementation	Mitochondria do not lie on a square lattice, so a more powerful model than the one we present can use randomly distributed nodes or use the microtubule network. A model that does not explicitly position nodes in space has been developed 58, but it does not consider diffusion on the network. The model in Ref. 58 of the mitochondrial network also shows percolation phenomena, and future work can extend the model by introducing a diffusing particle on the network and preferably introducing heterogeneity in fusion rates to represent heterogeneity in membrane potentials	This hypothesis suggests that the root mean squared distance travelled by mitochondrial proteins depends non-linearly on the connectedness of the network (network connectedness can e.g. be estimated by measuring the average length of a mitochondrial fragmented). Calculate the diffusion coefficient of proteins and the root mean squared distance travelled by these proteins while slowly changing fission or fusion rate
Increased ATP production caused by non-linear response of ATP synthesis rate to membrane potential	Numerous biophysical models of the respiratory chain in mitochondria have been developed (e.g. 59–61). Using this class of models to study respiratory chain and TCA cycle kinetics during fusion of two mitochondria can help in finding the existence and cause of increased ATP synthesis rate upon fusion	Measure Δ*ψ* (and preferably simultaneously ΔpH) of mitochondria before, during, and after fusion events. Measurements of Δ*ψ* during and after fission (but not fusion) events have been done before 24,62, and an asymmetry in potential is seen after fissioning 24. Whether this asymmetry is simply a reversion to a pre-existing asymmetry in potential before the preceding fusion event (fusion events are usually followed quite soon by fission events 24) should be determined. Alternatively, take snapshots of cells and quantify the amount of mitochondrial mass that is fused (by e.g. measuring the average mitochondrial size, which on average will be proportional to the level of connectivity in the cell) while also measuring [ATP]. Do this for many cells in order to search for a correlation between the amount of fused mitochondria and cellular [ATP]. Such correlations have been found before 8–11 but not without perturbing the cell. We propose to take advantage of natural fluctuations in connectedness. Alternatively, observe passive fluctuations in network size of mitochondria in single cells, while reading out [ATP]. As a final option, induce fragmentation while monitoring [ATP] and membrane potential during treatment
Improved bioenergetic control and energy production through Ca^2+^ signalling	Metabolic control analysis (MCA) can be used to predict how sensitive ATP production is to changes in activities of mitochondrial enzymes provoked by calcium differences. MCA has been used to study OXPHOS and glycolysis 63–67 and the TCA cycle 68. It was shown that *α*- ketoglutarate dehydrogenase and isocitrate dehydrogenase had 70 and 23% control over respiration, respectively 68. This result, however, is only true for specific conditions. To make physiologically relevant predictions, these models can be integrated into biophysical models of the TCA cycle and respiratory chain. Ordinary differential equation-based biophysical models linking calcium and mitochondrial physiology can also be probed to explore this relationship 56	Prepare two populations of cells, one wild-type and one with more fused mitochondria. Then stimulate [Ca^2+^]_cytoplasm_ while also measuring oxygen consumption of the populations. Compare the change in oxygen consumption induced by increased [Ca^2+^]_cytoplasm_ between the two populations of cells, to find a relationship between connectedness of the mitochondrial network and calcium-stimulated respiration. Alternatively, look at the distribution of [Ca^2+^]_matrix_ in single cells of both populations after stimulation of [Ca^2+^]_cytoplasm_, to check whether this distribution is more homogeneous in cells with more fused mitochondria (see Fig. 4)
Increased buffering against perturbations	Biophysical models of the mitochondrion that already exist (including, e.g. 69–71) can be used to check whether an increase in size indeed dampens fluctuations in membrane potential. However, one must be careful to use models that define the flux of ions into the mitochondrion to be proportional to surface area. The model presented in Ref. 59, for example, defines flux to be proportional to mitochondrial volume, and this model thus cannot be used to check the hypothesis discussed here.	While looking at (and quantifying) natural fluctuations in the size of a mitochondrial filament, measure fluctuations in membrane potential (and preferably also fluctuations in ΔpH) of this filament, to check whether larger filaments have smaller fluctuations. Alternatively, increase the permeability of the mitochondrial inner membrane in cells with fused mitochondria, and cells with fragmented mitochondria. Then measure the effect of this change in permeability (and thus biochemical fluctuations) on Δ*ψ* and ΔpH in both situations.

For each hypothesis discussed in the main text, we suggest further modelling approaches and experiments that will help test the hypothesis. Most of the experimental tests suggested will be possible with the tools available today. In this article we consider several hypotheses for an increase in [ATP] in highly fused mitochondrial states; in this table we only discuss hypothesis (iv): the others are discussed in sections S2.2.1, S2.2.2 and S2.2.3.

One immediate consequence of a mathematical perspective on mitochondrial fusion is the observation that, for fused states to be physiologically beneficial, a non-linear relationship between a mitochondrial property and cellular functionality must be involved. In other words, a fused mitochondrion must be of more use to the cell than the sum of the individual pre-fused mitochondria. Suppose *x* denotes some extensive property of a mitochondrion and *f *(*x*) describes how useful this mitochondrion is to the cell. Then, if there is another mitochondrion with property *y* and ‘usefulness’ *f *(*y*), we expect:

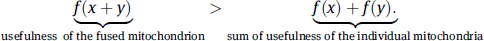


In other words, the function *f*, relating some aspect of mitochondrial size to ‘usefulness’, must be non-linear. Somehow fusion into networks must create ‘extra usefulness’ to the cell; the network structure must allow for, or give rise to, options that were not available before. In some of the hypotheses discussed, each individual fusion event benefits the cell; in others, a benefit only arises once sufficiently many mitochondria have fused to form an interconnected network.

### Selective fusion creates the possibility of quality control without needing selective mitophagy

#### Summary

Mitochondrial quality control is the process that maintains a healthy mitochondrial population by identifying and degrading dysfunctional mitochondria 24,72, degrading damaged mitochondrial components 73 and transporting damaged components out of the mitochondrion 74,75. Three mechanisms contributing to this control are fusion, fission and mitophagy. To ensure that a degradation bias is present (dysfunctional mitochondria should be more likely to be degraded than healthy mitochondria), selection occurring in one of these mechanisms is sufficient. We argue that selective fusion combined with non-selective fission and non-selective mitophagy leads to the desired biased degradation and that in fact there are two levels of quality control: (i) control on the level of the mitochondrial network (non-selective fission and selective fusion) that does not require mitophagy and which we call ‘blind surveillance’; and (ii) control on the level of the cell using non-selective mitophagy. In this section, our terms ‘selective’ and ‘non-selective’ refer to selection based on mitochondrial function; we assume that mitophagy always selects on mitochondrial size (i.e. only small mitochondrial fragments are targeted for mitophagy). Size-dependent mitophagy is the source of non-linearity in this model.

Blind surveillance consists of two parts. The first part involves random (blind) fission events. By random we mean that these events are not selective, and therefore a functional mitochondrion is just as likely to become fragmented as a dysfunctional one. The second part is the surveillance, which involves selective fusion events: a functional mitochondrion is more likely to fuse 24. As a consequence, a functional mitochondrion will, on average, stay fragmented for a shorter period of time than a dysfunctional mitochondrion. With blind surveillance present, mitophagy does not need to be selective because any isolated mitochondrion is more likely to be dysfunctional than healthy and, therefore, even if mitophagy is non-selective, a bias towards degrading dysfunctional mitochondria is automatically established. The strength of this effect is increased in cases of higher mitochondrial fusion (see section S1.1).

Having dysfunctional mitochondria separated from the fused network is an advantage on its own, because a dysfunctional mitochondrion fusing with an already fused and functional network may have negative effects that are not present if the mitochondrion in question remains isolated. Blind surveillance thus forms the a first level of quality control (it separates dysfunctional mitochondria from the network by using non-selective fission and selective fusion), and mitophagy forms the second level by eliminating fragmented mitochondria (which are more likely to be dysfunctional – hence even non-selective mitophagy eliminates more dysfunctional than functional mitochondria).

#### Experimental support

Selection on the level of fusion (mitochondria with low Δ*ψ* are less likely to fuse 28,29,24) and mitophagy 76–79 is supported by observation. A possible mechanism for selective fusion is the increased processing of OPA1 by OMA1 when Δ*ψ* is low 80,81, which then results in a lower fusion probability. Selective elimination of damaged mitochondria has been observed 82,83, although the precise mechanisms are still incompletely understood 84.

#### Coarse-grained quantitative model

Mathematical models of mitochondrial quality control have been constructed previously 85–87 and show that fission, selective fusion and selective autophagy together increase mitochondrial functionality. To address the specific role of network state, we construct a simple model of ordinary differential equations based on populations of healthy and dysfunctional mitochondria (for details see section S1.1). Assumptions made in this model are that only fragmented mitochondria can be removed by mitophagy (which causes the non-linearity), the total number of mitochondria is constant, and spatial distributions of mitochondria are ignored.

If the rate at which healthy mitochondria become dysfunctional is particularly slow, then the model predicts a steady state where all the dysfunctional mitochondria have been removed by non-selective mitophagy. If healthy mitochondria are allowed to become damaged, then steady states with both functional and dysfunctional mitochondria exist. When fusion rate is increased, the steady state fraction of functional mitochondria increases. We thus quantitatively illustrate that large-scale selective fusion, without need for selective mitophagy or fission, has a beneficial effect on cellular mitochondrial populations.

#### Limitations/Critique

Autophagy is required for this mechanism to function, although this assumption also underlies other quality control mechanisms. Our model makes several assumptions (mentioned above). A possibility for future work is to relax these assumptions (by e.g. including spatial distributions of mitochondria 86).

### Small changes in fusion state can cause large changes in complementation rate

#### Summary

Large-scale fusion facilitates sharing of mitochondrial machinery, as this machinery moves through the mitochondrial network. Increased sharing may equilibrate concentrations of nuclear encoded proteins to enable better control over mitochondria 39, promote coordinated behaviour between mitochondria to synchronize gene expression 1,88 or improve the health of the mitochondrial population by allowing mitochondria to complement each other’s deficiencies 89,90 (functional complementation).

The effect of increased fusion (or decreased fission) on diffusion rate is most important for fast-diffusing species because they will have their diffusion rate capped by fission and fusion rates. By diffusion rate we mean the effective rate of diffusion through the mitochondrial network, which will depend on fission and fusion rates. As the network becomes more interconnected, a fast-diffusing species will rapidly explore the new available spaces. Small increases in *p* (i.e. small increases in network connectivity may lead to large, non-linear, increases in diffusion rate, especially near the percolating value *p*_c_. This is because when *p* > *p*_c_ it suddenly becomes possible to diffuse from one extreme end of the cell to the other end, whereas when *p* < *p*_c_ the distance that can be travelled is much more restricted. A ‘switch-like’ response is thus present for fast-diffusing species. Slow-diffusing species will locally be exposed to a relatively rapidly fluctuating network structure; thus, their diffusion rate increases linearly with fusion level, meaning that there is no ‘switch-like’ response.

#### Experimental support

Functional complementation through fusion has been observed in numerous experimental studies 40,89,90, indicating that fused mitochondria do exchange contents. As mentioned before, the effect of increased connectedness of mitochondria is likely to be largest for fast-diffusing species. We therefore compare diffusion constants of several mitochondrial proteins (and mtDNA) in Table S3.

#### Coarse-grained quantitative model

We can study the diffusion of species along mitochondrial networks by simulations in which the network is represented by a 2D or 3D lattice. Mitochondria are represented by the nodes in the network, and they can be connected through the edges. The edges can flicker ‘on’ and ‘off’, representing fusion and fission events respectively, with rates *λ*_fus_ and *λ*_fis_. In this model, each mitochondrion has the potential to fuse with any of its direct neighbours independently, and does so with rate *λ*_fus_ (similarly for fission). The previously-defined value *p* broadly determines how likely it is that an edge is ‘on’. If the hopping rate for molecules moving between adjacent points on the network is given by *λ*_dif_, and particles are restricted to move only along ‘on’ edges, then the average time it takes a particle to diffuse across a certain distance can be calculated in dynamic hyperfused (*p*_c_ < *p* < 1), mesofused (0 < *p* < *p*_c_) and microfused (*p* ≈ 0) states (see section S1.2). Our coarse-grained model makes several assumptions, including constant mitochondrial mass (corresponding to a short timescale), an absence of mitophagy and a lattice topology (Ref. 58 provides an alternative approach).

**Figure 2:**
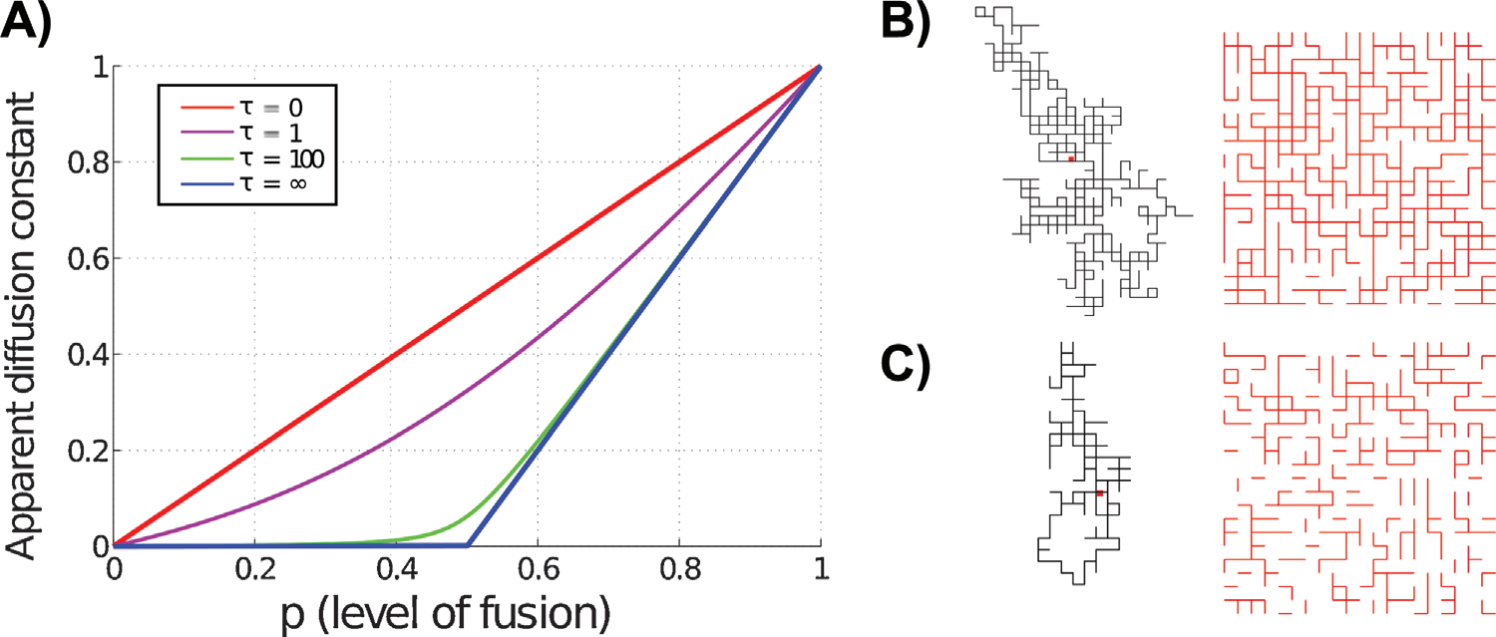
Apparent diffusion coefficient depends non-linearly on degree of fusion. An abrupt change in diffusion rate can occur with only a small change in fusion rate. A: This figure shows the diffusion constant of a particle diffusing on a 2D fluctuating lattice as a function of *p* (the fraction of present bonds) and *τ* (the relaxation time of the fluctuating bonds). If *τ* = ∞, the bonds are static; conversely, *τ* = 0 corresponds to the limit of very fast fluctuating bonds. B: A single trajectory of a diffusing particle (on the left) and a lattice snapshot for *p *= 0.6 and *τ* = 100 (on the right). The red dot in the trajectory marks the starting point of the particle. Existing bonds in the lattice snapshot are shown in red. C: Trajectory and lattice snapshot for *p* = 0.4 and *τ* = 100. Figure B and C show that (for *τ* = 100) increasing the value of *p* from 0.4 to 0.6, results in a more connected network and less restricted diffusion, as is also suggested in Fig. A which indicates a rather abrupt increase in effective diffusion rate around *p* = 0.5.

In Fig. 2A, it is shown how the apparent diffusion coefficient varies with *p* for different values of *τ*, where *τ* is the relaxation time of the fluctuating bonds 91,92 and is defined as 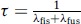 (a lower *τ* means that bonds flicker more frequently between the states ‘on’ and ‘off’). This figure is based on a mean field approximation, and similar results are produced by our agent-based stochastic model. In static percolation (*τ* = ∞), an abrupt change in diffusion rate is present at *p* = 0.5 (in 2D, assuming a square lattice), but when bonds fluctuate in time, the diffusion rate changes less abruptly. For a given *p*, letting bonds fluctuate faster has the effect of increasing the diffusion rate, and this effect is largest around *p* = 0.5. The simple model shows that a microfused state results in a smaller diffusion rate than a hyperfused state, even if the microfused state has very frequent fusion and fission events. Even slowly-varying hyperfused networks thus afford more facility to spread elements throughout the cell, confirming that a hyperfused morphology confers a mixing advantage for proteins.

#### Limitations/Critique

Complementation may be sufficiently achieved without a need for large-scale fusion: short ‘kiss and run’ events are claimed to be responsible for most mixing because of their high frequency 43. In the spirit of ‘back-of-the-envelope’ calculations in biology 93, we use rough estimates to assess the feasibility of equilibration. The duration of association of these fusion events approximately ranges from 4 seconds to 5 minutes, with a mean of 45 seconds 43. The diffusion coefficient of Green Fluorescent Protein was measured to be 20–30 µm^2^/s 94,95, meaning that in 45 seconds a distance of about 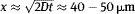 can be travelled, which is more than enough to accomplish equilibration. However, even though complementation of matrix components is feasible, kiss and run events may be too short to allow for sharing of membrane proteins and nucleoids.

### Fusion increases ATP synthesis

#### Summary

Mitochondrial fusion may increase ATP production, through one or more of several possible mechanisms. We suggest the following possible mechanisms of particular interest: (i) higher [ATP] is caused by fusion-induced changes in inner membrane shape; (ii) higher [ATP] is caused by fusion-induced decreases in proton leak; (iii) higher [ATP] is caused by fusion-induced decreases in mitochondrial degradation; (iv) higher [ATP] is caused by the non-linear response of ATP synthesis rate to membrane potential. (i–iii) are discussed in more detail in the SI. We note that an assumption made in all four hypotheses is that fusion causes in higher [ATP]. However, it remains to be determined whether mitochondrial fusion is the cause of an observed increase in [ATP], or whether hyperfusion and high ATP concentrations have a common cause, e.g. a recent study suggested that an increase in ATP production may precede mitochondrial fusion 96.

#### Experimental support

Several studies suggest that hyperfusion increases ATP levels and mitochondrial respiratory capacity 8–11. In nutrient-rich environments mitochondria tend to be fragmented, whereas under starvation they are observed to form elongated networks 9,12, which can be interpreted as an attempt to enhance energy production in challenging environments. Hyperfusion is also observed at the G1/S transition, before energetically costly DNA replication 8. It is worth noting that in hyperfused states, concentrations of cellular ATP – not just the rate of ATP production – were measured to be higher 10. These increased concentrations suggest a physiological role for fusion beyond that of meeting extra ATP demand. This role could be to act as a cellular ‘accelerator pedal’, because an increase in ATP/ADP ratio in the cell has the consequence that many reactions will go faster 97,98. Further support for specific hypotheses is described in sections S2.2.1, S2.2.2 and S2.2.3.

#### Coarse-grained quantitative model

We focus mathematically on hypothesis (iv) which we have not seen discussed elsewhere. We first consider how the membrane potentials of two individual mitochondria Δ*ψ*_1_, Δ*ψ*_2_ are related to the overall membrane potential of their fused product Δ*ψ*_1+2_. A simple model is that the fused product inherits the arithmetic average of the two individuals, i.e. 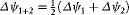. However, physical calculations based on considering charged capacitors suggest a range of possibilities depending on modelled cristae structure 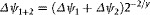 where 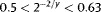 (see section S1.4). We do not claim that this capacitor model is a particularly realistic one (in particular, it does not necessarily capture the behaviour of mitochondria fusing in chains), but we use it to give a quantitative example of the possibility of non-averaging potentials when two charged objects are combined.

**Figure 3:**
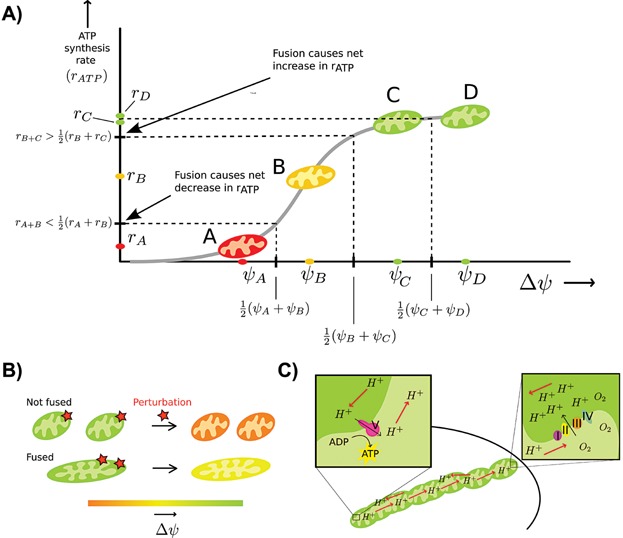
A: The effect of fusion on rate of ATP synthesis depends on the magnitude of the potentials of the pre-fusing mitochondria. In this figure, *r*_i_ denotes the ATP synthesis rate (*r*_ATP_) for mitochondrion i and *r*_(i + j)_ denotes the rate for the fused product of mitochondria i and j. For simplicity we assume equal mitochondrial size; our results still hold when this assumption is relaxed. Because of the non-linear dependence of *r*_ATP_ on Δ*ψ*, if two mitochondria in the exponential regime fuse (i.e. mitochondria A and B), then averaging their potentials upon fusion causes the net ATP synthesis rate to decrease. This is because 2 *r*_(A + B)_ < *r*_A_ + *r*_B_ (the fused mitochondrion is twice as large as the pre-fused mitochondria and we thus effectively have two mitochondria post-fusion, each with rate *r*_(A + B)_). In the plateau region, *r*_ATP_ does not depend on Δ*ψ*, so there will most likely be no Δ*ψ* induced change in *r*_ATP_ if two mitochondria in this regime fuse (e.g. mitochondria C and D). If a mitochondrion from the exponential regime fuses with one from the plateau (i.e. mitochondria B and C), net *r*_ATP_ increases because 2 *r*_(B + C)_ > *r*_B_ + *r*_C_. B: Mitochondrial fusion buffers fluctuations in membrane potential. Opening of the mitochondrial permeability transition pore or changes in ion leakage can lead to depolarizations of mitochondria. These perturbations to the membrane potential will have less effect when mitochondria are fused because their bigger size makes them more robust. C: Mechanism of mitochondrial power cabling. Oxygen concentrations are higher at the periphery of the cell and mitochondria positioned here will pump protons out of their matrix. In the core of the cell, if ATP is required, the ATP synthase will pump protons into the matrix. A proton gradient establishes itself along the mitochondrial cable and protons diffuse thereby transmitting chemical potential. This is the main idea of mitochondrial power cabling; a replacement of diffusion of ATP or oxygen through the cytoplasm by proton movement along mitochondrial filaments, which may result in an increased speed of energy transmission.

Fusion has a clear advantage in the case that 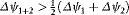. We argue that if potentials do average (i.e. 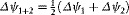), it is still possible that total ATP synthesis rate increases (or decreases) upon fusion. This advantage is due to a sigmoidal dependence of ATP synthesis rate (*r_ATP_*) on Δ*ψ* 99,100 (see Fig. 3A). Referring to the illustration in Fig. 3A, fusion of mitochondria B and C causes an increase in *r_ATP_* (i.e. *r_ATP_* is larger post-fission than pre-fission) whereas fusing A and B causes a decrease in *r_ATP_* and fusion of C and D leaves *r_ATP_* approximately constant. The effect of fusion on rate of ATP synthesis therefore depends on the magnitude of the potentials of the pre-fusing mitochondria. Having mitochondria like A fusing to others causes net decreases in energy production, and this is a possible reason for why mitochondria with low Δ*ψ* have a lower fusion probability 28,29,24.

#### Limitations/Critique

The causal relationship between higher ATP levels and mitochondrial fusion is still incompletely understood, and it may be possible that fusion is the effect of high [ATP] instead of the cause. A recent study showed that the rate of inner membrane fusion was closely correlated with oxygen consumption, and that this rate increased during OXPHOS stimulation as the result of increased OPA1 cleavage by Yme1l 96. Also, in the model discussed above we have considered the role of Δ*ψ* in ATP synthesis rate, whereas actually the proton motive force (defined as 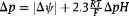) drives ATP production 99,100. The region of the ATP synthesis sigmoid at which mitochondria lie is not yet experimentally clear. Further critique of specific hypotheses is described in sections S2.2.1, S2.2.2 and S2.2.3.

### Fusion affects bioenergetic control and total energy production through calcium signalling

#### Summary

Fusion averages out calcium concentrations across the mitochondrial network, giving rise to two advantages: (i) calcium concentrations are placed in an intermediate regime where small changes have pronounced effects on enzymatic rates, and thus tight bioenergetic control of the mitochondrial population is facilitated; and (ii) the amount of mitochondrial volume that experiences a rise in [Ca^2+^]_matrix_ is increased, hence boosting total energy production.

**Figure 4:**
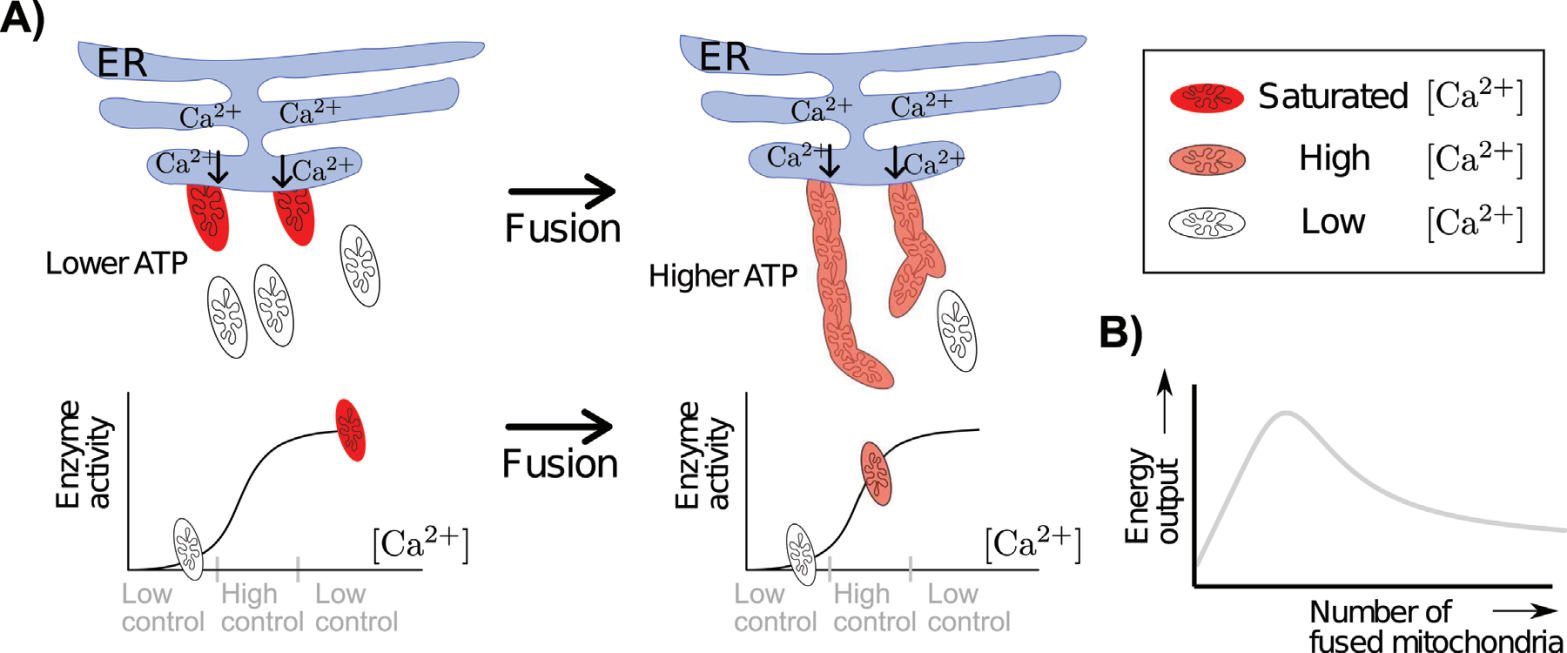
Fusion may increase controllability of TCA cycle activity and total energy production. A: If only mitochondria in contact with the ER easily take up calcium, then in a fragmented state only few mitochondria will have high (saturated) [Ca^2+^]_matrix_ and most will have low [Ca^2+^]_matrix_. The bottom figure indicates the position of the mitochondria on the sigmoid relating calcium concentration to enzymatic activity. If mitochondria fuse, [Ca^2+^]_matrix_ averages out, which moves the previously saturated mitochondria down the sigmoid and also moves the mitochondria which previously had very low [Ca^2+^]_matrix_ up the sigmoid with the result of increasing total enzyme stimulation. When even more mitochondria fuse, total enzyme stimulation will drop again because the mitochondria moved too far down the sigmoid. B: As the number of mitochondria fused to a mitochondrion close to the ER increases, total energy output will first increase and then decrease again because [Ca^2+^]_matrix_ is now so diluted as to reach the low enzyme activity regime. This plot is a schematic illustration of principle, details are given in section S1.5.

Calcium is known to stimulate certain enzymes of the TCA cycle 101–103, which in turn influences the rate of ATP production. The dependence of TCA cycle activity on calcium is sigmoidal 104, which means that the cell has maximal control over this activity when [Ca^2+^]_matrix_ is in the steeply rising part of the sigmoid curve, as indicated in Fig. 4A. If [Ca^2+^]_matrix_ is high enough to reach the plateau region of the sigmoid, the enzymes are saturated. Only mitochondria located near microdomains of high [Ca^2+^] (e.g. ER calcium channels) are thought to take up calcium efficiently 105,106. The calcium concentration in these mitochondria rises significantly during ER depletion or calcium entry into the cell. This rise in [Ca^2+^]_matrix_ can be such that the saturation level is reached, and increasing [Ca^2+^]_matrix_ further has no effect on enzyme activity. Because fusion averages out concentrations inside the matrix, fusing a calcium-saturated mitochondrion with one with low [Ca^2+^]_matrix_ has the effect of shifting [Ca^2+^]_matrix_ towards the more controllable region. Mitochondria far from high [Ca^2+^] microdomains with lower [Ca^2+^]_matrix_ increase stimulation of their TCA cycles by fusing with a mitochondrion with high [Ca^2+^]_matrix_. Fusion therefore increases the amount of mitochondrial volume affected by a calcium signal, which subsequently increases total energy production.

#### Experimental support

The idea that fusion increases the number of mitochondria that experiences a given calcium signal in the cell has been suggested before in Ref. 107.

#### Coarse-grained quantitative model

We focus on the uptake of calcium from the ER as the most important influence on mitochondrial calcium levels, assuming that without fusion, the mitochondria near ER calcium channels have saturated calcium-dependent TCA cycle enzymes, and the mitochondria far from the ER or plasma membrane have low [Ca^2+^]_matrix_ (as in Fig. 4A). The first few fusion events will increase total enzyme stimulation (and therefore ATP production) because the mitochondria fusing to the calcium saturated ones experience a significant increases in [Ca^2+^]_matrix_ and enzyme activation, whereas the saturated mitochondria experience little change in enzyme activation, despite their drop in [Ca^2+^]_matrix_ (because of the sigmoidal dependence of enzyme activity on [Ca^2+^]). Adding more and more mitochondria to the fused chain will ultimately lead to a decrease in enzyme stimulation (because [Ca^2+^]_matrix_ will reach the lower part of the sigmoid), meaning that there exists a certain number of fused mitochondria that leads to maximal enzyme activation. This number depends on the amount of calcium released by the ER, the number of mitochondria near the ER, the basal [Ca^2+^]_matrix_ present in mitochondria far from the ER, the exact shape of the sigmoid, and, furthermore, relies on the assumption that when many mitochondria are fused, [Ca^2+^]_matrix_ becomes so diluted as to reach the low enzyme activity regime. There will of course be many other factors that influence this ‘optimal number of fused mitochondria’ (e.g. the ratio of phosphate bound calcium to free calcium): we merely present the basic idea, which is also shown in Fig. 4B (for details see section S1.5).

#### Limitations/Critique

This hypothesis is based on several assumptions that are debated in the literature. While several studies show that higher calcium concentrations lead to increases in NADH production and oxygen consumption 68,108,109, mathematical modelling 56 and uniporter studies 110 suggest that calcium perturbations may have little effect on respiration. Other studies have observed that calcium only has an effect on NAD(P)H concentrations in glucose-stimulated conditions 111, or that only a single TCA enzyme is controlled by calcium 104. The mechanisms of calcium uptake that are mainly used, the dependence of these mechanisms on calcium concentrations, and the number of sites close to sources of calcium are also debated 105,112 (for details, see section S1.6). Mitochondria have been observed to experience calcium transients, meaning that they extrude calcium quite rapidly after having absorbed it 53 and the simple model we discuss in section S1.5 may therefore only be valid on short timescales. Finally, calcium signalling linked to mitochondrial ultrastructure is by no means the only, or simplest, way in which the cell regulates its energy status.

### Fusion provokes state changes protective against perturbations

#### Summary

By changing their morphological state, mitochondria can make themselves less susceptible to perturbations (for example, fluctuations in electrochemical membrane potential). The fused neighbours of a mitochondrial element can act as a buffer for biochemical or physical fluctuations. If failure of individual mitochondria has severe consequences for the cell, this increased robustness through fusion will be beneficial when mitochondria are subject to perturbations.

#### Experimental support

There are some experimental indications that fused mitochondria are better protected against stress 113. This hypothesis could account for why hyperfusion occurs during stress 10, and may also be an explanation as to why fusion protects the cell against apoptosis 10,13–16 (or at least delays apoptosis 114), because prior to cytochrome c release (which induces apoptosis) remodelling of the cristae structure has been seen as a cause of changes in membrane potential 115. If a larger network protects against fluctuations in membrane potential, it may be able to prevent cristae remodelling, thereby potentially delaying or preventing apoptosis.

In stressful conditions, it may thus be disadvantageous for the cell to have its mitochondria fragmented because all of them are vulnerable to membrane potential fluctuations. If the cell fuses its mitochondria, they become more robust to the fluctuations, which may prevent the failure of many individual mitochondria, and subsequent cell death.

#### Coarse-grained quantitative model

A biophysical calculation considering the change in membrane potential upon changes in permeability of the inner mitochondrial membrane (see section S1.7.1) suggests 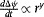 where *r* is the radius of the mitochondrion and 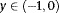 depends on the structure of the membrane (the more invaginated the membrane, the larger *y*). This result suggests that for large mitochondria, fluctuations of Δ*ψ* caused by transient perturbations will be of smaller magnitude. Fusing mitochondria thus protects them from perturbations, as illustrated in Fig. 3B. This model assumes spherical mitochondrial geometry. Small fragmented mitochondria are often seen to have a spherical shape and the fusion of two small spherical mitochondria may produce a mitochondrion that itself has an approximate spherical shape. The model discussed here may therefore be applied to fusion events involving small mitochondrial fragments.

We provide an alternative model that is independent of volume and surface area scaling and involves picturing mitochondria as individual agents coupled with spin-like interactions 116. This model, in which a mitochondrion prefers to be in a similar state as its neighbours, shows that groups of mitochondria are less likely to undergo a catastrophic loss of function than individuals (see section S1.7.2).

#### Limitations/Critique

Even though fluctuations in larger mitochondria (fused mitochondria) may be of smaller relative magnitude, they will occur more frequently because of the larger surface area which increases the probability that, for example, a pore opens or an ETC component fails. Stress does not always lead to a fused mitochondrial state, but can also lead to mitochondrial fragmentation 117,118. It can be that the level of stress is important, and that too much stress leads to fragmentation and subsequent apoptosis.

## Conclusions

The function of mitochondrial networks is currently unclear, suggesting that new research strategies may be of use. We have shown that ideas from physics and mathematics provide a framework to suggest and critically evaluate hypothesised functions. Among the insights we underline from this work are the possibilities that ‘blind surveillance’ through selective fusion alone leads to an increase in mitochondrial quality control; that increased fusion may have non-linear effects on the diffusion rate of proteins; that the effect on membrane potential of fusion may be more complicated than a simple averaging; and that fusion can act to dampen biochemical fluctuations.

Which hypothesis is most likely to be true? Three hypotheses that we find attractive are (i) increased robustness; (ii) blind surveillance; and (iii) increased ATP production through non-linear dependence of ATP flux on the properties of fusing mitochondria. Hypothesis (i) suggests a reason why mitochondrial morphology is dependent on cellular stress, which seems to be the main regulator of mitochondrial morphology. Hypothesis (ii) also links fusion with oxidative stress, because in stress conditions improved quality control is beneficial. Hypothesis (iii) provides intuitive mechanisms by which fusion may improve the energetic status of the cell, compatible with a large amount of evidence that mitochondrial structure is correlated with bioenergetic capabilities of the cell. We stress again the observation that with increased fusion, ATP concentration, rather than merely the rate of production and consumption of ATP, changes. This implies an acceleration of cellular processes and suggests that fusion serves as a cellular accelerator pedal. We cannot exclude the possibility that the central ‘purpose’ of network formation is not one of the currently considered hypotheses; but we anticipate that quantitative modelling approaches will also be powerful tools in analysing any future hypotheses.

The authors have declared no conflicts of interest.

## Abbreviations

ER: endoplasmic reticulum
ETC: electron transport chain
mtDNA: mitochondrial DNA
TCA cycle: tricarboxylic acid cycle

## Supporting Information

As a service to our authors and readers, this journal provides supporting information supplied by the authors. Such materials are peer reviewed and may be re-organized for online delivery, but are not copy-edited or typeset. Technical support issues arising from supporting information (other than missing files) should be addressed to the authors.
