# Supplementary material for: What is the function of mitochondrial networks? A theoretical assessment of hypotheses and proposal for future research
Source: Bioessays. 2015 Apr 3;37(6):687–700. doi: 10.1002/bies.201400188 (PMC4672710; doi:10.1002/bies.201400188)
Supplement: Supplementary file 1 [file bies0037-0687-sd1.pdf]

Supplementary Information

This Supplementary Information is split into three sections:

1. The first section contains quantitative and mathematical details of various models used in the main text. Several of these models follow the philosophy of 'back-of-the-envelope' calculations, rough estimations of quantities which are increasingly recognised as powerful tools to gain insights and intuition about the behaviour of biological systems [1, 2]. The models we use are:

   - Ordinary differential equation model for selection bias through selective fusion;
   - Model for diffusion on a lattice with fluctuating bonds;
   - ATP flux upon averaging membrane potentials;
   - Fusion of charged spherical capacitors;
   - Calcium stimulation and energy output of fused mitochondria;
   - Mechanisms of calcium uptake;
   - Fusion buffers fluctuations in membrane potential.

2. The second section contains descriptions of additional hypotheses mentioned in the main text:

   - A genetic reservoir created through fusion;
   - Fusion increases ATP synthesis through (i) inner membrane shape change; (ii) decrease in proton leak; (iii) decreases in mitochondrial degradation.
   - Fusion creates distributional 'power cables'
   - Meso- and hyperfusion have no function

3. The third section contains tables summarising quantitative and qualitative data on the following topics:

   - Observations of hyperfusion;
   - Mitochondrial morphology in different cell types;
   - Diffusion constants of mitochondria proteins;
   - Post-translational modifications of Drp1;
   - Additional functions of mitochondrial fission and fusion proteins.

1 Quantitative and mathematical models

1.1 Model for increased selection bias through selective fusion

We will show with a simple ordinary differential equation model that increased fusion or decreased fission strengthens the bias towards degrading dysfunctional mitochondria, an effect explained intuitively in section 3.1. We study the dynamics of four populations: i) healthy fused, ii) healthy fragmented, iii) dysfunctional fused, and iv) dysfunctional fragmented mitochondria whose concentrations are represented by $c_{gu}, c_{bu}, c_{gf}, c_{bf}$ respectively (the subscripts 'gf' and 'bu' stand for 'good fused' and 'bad unfused').

We assume that healthy mitochondria undergo fusion at a rate λ_{gf} while dysfunctional mitochondria fuse at rate λ_{bf}, where we encode selective fusion by assuming that $\lambda_{gf} > \lambda_{bf}$. The rate of fission is the same for healthy and dysfunctional mitochondria and is given by λ_f. We also assume that a mitochondrion cannot change its status from healthy to dysfunctional or vice versa (this assumption is relaxed later). New mitochondria are created by biogenesis and mitochondria are being removed by mitophagy. Let M and B denote the rates of mitophagy and biogenesis respectively. Our crucial assumption is that only fragmented mitochondria can be removed by mitophagy and we impose a constraint to keep the total number of mitochondria constant, i.e. $B(c_{gf} + c_{bf} + c_{gu} + c_{bu}) = M(c_{gu} + c_{bu})$. The dynamics of this system is given by the following equations:

$$
\begin{aligned}
\dot{c}_{gu} &= -c_{gu}\left(\lambda_{gf} + B\frac{c_{gf}+c_{bf}}{c_{gu}+c_{bu}}\right) + \lambda_f c_{gf} \\
\dot{c}_{bu} &= -c_{bu}\left(\lambda_{bf} + B\frac{c_{gf}+c_{bf}}{c_{gu}+c_{bu}}\right) + \lambda_f c_{bf} \\
\dot{c}_{gf} &= c_{gf}(B-\lambda_f) + \lambda_{gf} c_{gu} \\
\dot{c}_{bf} &= c_{bf}(B-\lambda_f) + \lambda_{bf} c_{bu}
\end{aligned} \tag{1.1}
$$

where we have eliminated M by using the above-mentioned constraint. This system of ordinary differential equations can be solved and the population of dysfunctional mitochondria $(c_{bf} + c_{bu})$ tends towards zero as can be seen in Figure 1. If fusion rate is increased, while increasing λ_{gf} more than λ_{bf}, then the population of dysfunctional mitochondria decreases faster. If fission rate is decreased, then the ratio $\frac{c_{bu}}{c_{gu}+c_{bu}}$ will be larger and the total population of dysfunctional mitochondria will decay slower (because of an increase in dysfunctional fused mitochondria). This means that if the cell wants to decrease the number of dysfunctional mitochondria as quickly as possible, increasing fusion rate would be the solution. Decreasing fission rate has the effect of keeping dysfunctional mitochondria in the total population for a longer time, but mitophagy events will remove fewer healthy mitochondria because of the increase in $\frac{c_{bu}}{c_{gu}+c_{bu}}$.

We note that, in reality, there will be a rate at which functional mitochondria become dysfunctional. This rate is very low if one only considers dysfunctionalities caused by mtDNA mutations, in which case the model described above may be used as an approximation; mitochondria with a certain mtDNA mutation will be removed from the network before the next mtDNA mutation occurs. However, mitochondria can become dysfunctional in other ways (e.g. depolarization events or oxidative damage), in which case the described model is not sufficient. Extra terms can be added to Equations (1.1) to account for the possibility that a 'good' mitochondrion becomes a 'bad' mitochondrion. These extra terms allow for the possibility of steady states with a nonzero populations size for all of the populations. If fusion rate is increased (while maintaining a constant ratio $\lambda_{gf}/\lambda_{bf}$), the ratio $\frac{c_{gf}+c_{gu}}{c_{bf}+c_{bu}}$ in steady state becomes larger (i.e. the higher fusion rate, the higher the fraction of functional mitochondria in steady state).

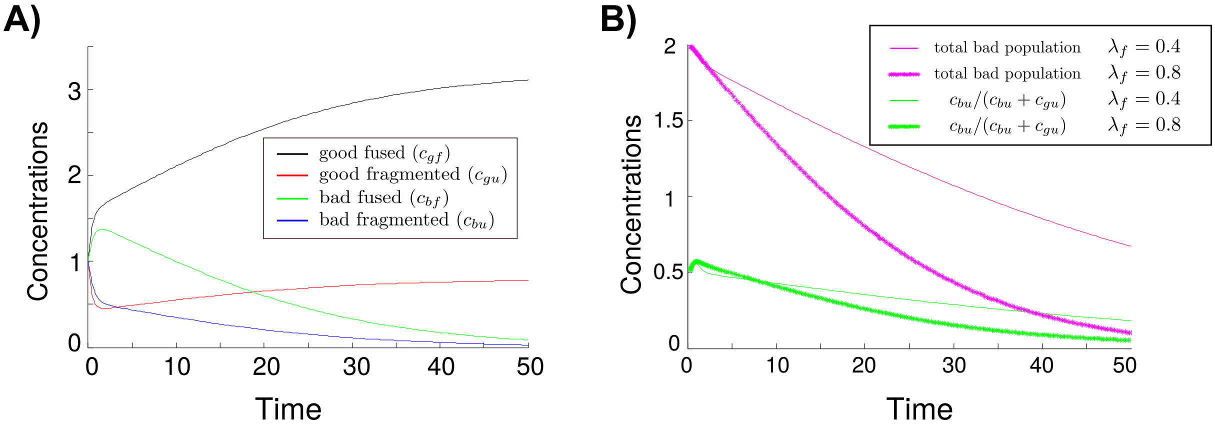

Figure 1: **Selective fusion on its own leads to a healthier mitochondrial population. A:** the trajectories of c_{gf}, c_{bf}, c_{gu} and c_{bu} are shown as a function of time. The parameters used in this figure are $\lambda_f = 0.8$, $\lambda_{gf} = 2$, $\lambda_{bf} = 1.3$ and $B = 0.3$. Over time only the healthy mitochondria survive because new mitochondria are being produced by biogenesis and a bias towards degrading dysfunctional mitochondria is present, even though the rate of mitophagy M is equal for healthy and dysfunctional mitochondria. This behaviour does not depend on the initial conditions of the system. **B:** The parameters are the same as in the A, except that for the dashed line the fission rate is decreased. One can see that the effect of a lower λ_f is that at any given time (except for very small times) the ratio $\frac{c_{bu}}{c_{gu}+c_{bu}}$ is higher and the total dysfunctional population $(c_{bu} + c_{bf})$ decays at a slower rate.

1.2 Model for diffusion on lattice with fluctuating bonds

Diffusion on lattices with 'flickering' bonds (bonds that can appear and disappear over time) has been studied in [3, 4], using the theory described in [5]. An analytical approach, taking into account the state of all the bonds, is intractable and therefore an effective medium approximation is used [3, 4]. The fluctuating lattice is approximated by a static lattice in which the transition rate to a nearest neighbour is the same for all nearest

neighbour pairs and is given by w_e (the effective transition rate), satisfying

$$\frac{p - w_e}{p_c(1 - w_e)} = 1 - \frac{1}{1 + n_c w_e \tau} G\left(0, 1 - \frac{1}{1 + n_c w_e \tau}\right) \tag{1.2}$$

where p is the fraction of bonds that are present, n_c is the number of nearest neighbours per site, $p_c = \frac{2}{n_c}$, τ is the characteristic relaxation time of the fluctuating bonds, and $G(0, z)$ is the appropriate lattice Green's function evaluated at the origin [3], which in the case of our 2D square lattice model is

$$G(0, z) = \frac{2}{\pi} K(z^2) \tag{1.3}$$

where K is the complete elliptical integral of the first kind.

This approximate model is equivalent to random diffusion on a lattice with a hopping rate given by w_e and therefore w_e can be interpreted as the diffusion coefficient D. Equation (1.2) is used to generate Figure 2A in the main text.

1.3 Averaging potentials upon fusion leads to decreases in ATP flux because of non-linearities

Here we present some arguments to explain why in certain conditions averaging potentials upon fusion may be disadvantageous and may lead to a decrease in rate of ATP production, as claimed in Figure 3A of section 3.3. For now we only focus on one variable on which the ATP synthesis rate is dependent: the membrane potential $\Delta\psi$. The dependence of the ATP synthesis rate (r_{ATP}) on $\Delta\psi$ is sigmoidal [6, 7]. If the exponential range of this sigmoidal function lies within the physiological range of $\Delta\psi$, then increases in the average $\Delta\psi$ in the cell lead to exponential increases in ATP synthesis rate. What happens to the membrane potential if two mitochondria fuse? If it averages then $\Delta\psi$ contributes to a decrease in r_{ATP}, whereas if the fused membrane potential becomes slightly higher than the average, then it is possible that r_{ATP} increases. We will show this below.

Let the flux through an ATP synthase, as function of the membrane potential, have the following exponential form

$$J_{F1} = Ce^{A\Delta\psi + B} \tag{1.4}$$

which is based on the ATP synthase flux given in [8]. We treat A, B and C as constants, ignoring their slight dependence on the chemical environment [8], because we are only interested in the effect that $\Delta\psi$ has on the flux. A value for the 'constant' A (which we will need later) can be found in [8] and is given by $A = \frac{n_a F}{RT} \approx 0.1\text{mV}^{-1}$ where $n_a \approx 3$ (the number of protons needed to create one ATP molecule), $T = 37°$C, F is the Faraday constant and R is the gas constant.

Suppose now we fuse two mitochondria with potentials $\Delta\psi_1$ and $\Delta\psi_2$ into a mitochondrion with potential $\Delta\psi_{1+2}$. Besides the difference in membrane potential, we assume that the two pre-fused mitochondria are identical, i.e. they have the same volume, the same number of cristae and the same number of ATP synthases embedded in their membrane. The total flux of ATP generated by these two mitochondria in the initial (pre-fusion) state is now given by

$$J_i = N_{F1}\left(Ce^{A\Delta\psi_1 + B} + Ce^{A\Delta\psi_2 + B}\right) \tag{1.5}$$

where N_{F1} is the number of ATP synthases that these mitochondria each have.

If we average the potential upon fusion, i.e. $\Delta\psi_{1+2} = \frac{1}{2}(\Delta\psi_1 + \Delta\psi_2)$ then the flux in the final (post-fusion) state is given by

$$J_f = 2N_{F1}(Ce^{A\frac{1}{2}(\Delta\psi_1 + \Delta\psi_2) + B}) \tag{1.6}$$

where the factor of two appears because the number of ATP synthases in this fused mitochondrion is twice that of the pre-fused mitochondria. The ratio of the final flux over the initial flux is given by

$$\begin{aligned} \frac{J_f}{J_i} &= \frac{2e^{A\frac{1}{2}(\Delta\psi_1 + \Delta\psi_2)}}{e^{A\Delta\psi_1} + e^{A\Delta\psi_2}} \\ &= \frac{2e^{\frac{A}{2}(\Delta\psi_2 - \Delta\psi_1)}}{1 + e^{A(\Delta\psi_2 - \Delta\psi_1)}} \end{aligned} \tag{1.7}$$

Equation (1.7) is plotted as the black line in Figure 2, which shows that *averaging of pre-fused potentials decreases total ATP synthase flux.*

To reconcile this result with observed increases in ATP synthase flux upon fusion, we investigate what happens when $\Delta\psi_{1+2}$ becomes slightly higher than the average, i.e. $\alpha > 0.5$. The ratio of final to initial flux is now given by

$$\frac{J_f}{J_i} = \frac{2e^{\alpha x + A\Delta\psi_1(2\alpha-1)}}{1+e^x} \tag{1.8}$$

where $x = A(\Delta\psi_2 - \Delta\psi_1)$. Using $A \approx 0.1$ mV^{-1} and fixing $\Delta\psi_1$ at 180 mV, Equation (1.8) can be plotted for different values of α. A plot for $\alpha = 0.501$ is shown as the blue line in Figure 2. Choosing a different value for $\Delta\psi_1$ leads to similar results. There is an interplay between the value of α and magnitude of the difference between the potentials of fusing mitochondria for which fusion is beneficial.

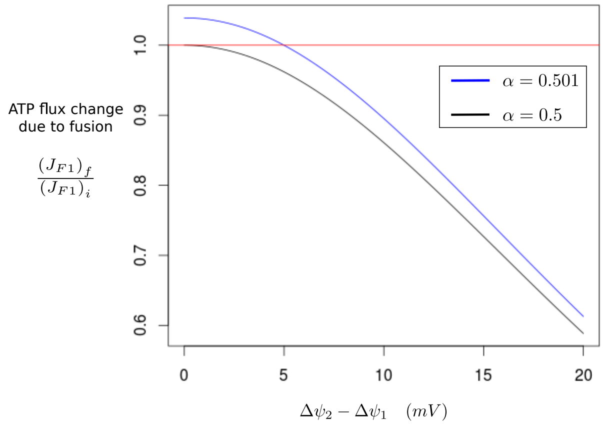

Figure 2: **Averaging membrane potential upon mitochondrial fusion leads to a decrease in ATP synthesis rate.** Let $\Delta\psi_{1+2} = \alpha(\Delta\psi_1 + \Delta\psi_2)$. If membrane potential averages upon fusion (black line, $\alpha = 0.5$) the post-fused ATP flux is lower than the pre-fused ATP flux. To get an increase in ATP flux we can slightly increase the mean potential (blue line, $\alpha = 0.501$).

1.4 Fusion of charged spherical capacitors

Here we explain how fusion of two charged objects can lead to non-averaging potentials, as claimed in section 3.3. Suppose we have two charged shells, a small one with radius r_{in} and a bigger one with radius r_{out}. The spheres are assumed to have opposite charges, i.e. one has charge Q and the other charge $-Q$. Applying Gauss' law, the potential difference between the shells is given by

$$V = \frac{Q}{4\pi\epsilon} \cdot \frac{r_{out} - r_{in}}{r_{in} r_{out}}. \tag{1.9}$$

where ϵ is the dielectric permittivity of the medium between the charged spheres. Because the capacitance C is given by $C = \frac{Q}{V}$, Equation (1.9) lead to

$$C = \frac{4\pi\epsilon r_{in}(r_{in} + d)}{d} \tag{1.10}$$

where we have denoted the difference between the inner and outer radius by d.

We now consider a 'fusion' of these two capacitors, such that total volume and charge is conserved. Denote by r_i and Q_i respectively the radius and charge of capacitor i, and label the initial two capacitors 1 and 2, and the resultant fused capacitor 3. If we maintain a spherical structure in the resulting, fused capacitor, the conservation of volume translates into the constraint that $r_3^3 = r_1^3 + r_2^3$. Conservation of charge gives $Q_3 = Q_1 + Q_2$. The capacitance of the fused capacitor is then given by

$$\begin{aligned} C_3 &= \frac{4\pi\epsilon r_3(r_3 + d)}{d} \\ &= \frac{4\pi\epsilon(r_1^3 + r_2^3)^{1/3}\left[(r_1^3 + r_2^3)^{1/3} + d\right]}{d}, \end{aligned} \tag{1.11}$$

and the potential V_3 can now be calculated as follows

$$\begin{aligned} V_3 &= \frac{Q_1 + Q_2}{C_3} \\ &= \frac{V_1 C_1 + V_2 C_2}{C_3} \\ &= \frac{V_1 r_1 (r_1 + d) + V_2 r_2 (r_2 + d)}{(r_1^3 + r_2^3)^{1/3}\left[(r_1^3 + r_2^3)^{1/3} + d\right]}. \end{aligned} \tag{1.12}$$

For a mitochondrion, the thickness of the inner membrane is about 5 nm and is two orders of magnitude less than the value for r_{in} which can be around 0.5 µm. Using $d \ll r_i \, \forall i$, Equation (1.12) becomes

$$\begin{aligned} V_3 &= \frac{V_1 r_1^2 + V_2 r_2^2}{(r_1^3 + r_2^3)^{2/3}} \\ &= \frac{V_1 A_1 + V_2 A_2}{(A_1^{3/2} + A_2^{3/2})^{2/3}}, \end{aligned} \tag{1.13}$$

where $A_i = 4\pi r_i^2$ is the surface area of capacitor i. In the case that the two fusing capacitors have the same surface area (i.e. $A_1 = A_2$) this reduces to

$$V_3 = (V_1 + V_2) 2^{-2/3} \approx 0.63(V_1 + V_2) \tag{1.14}$$

This means that the voltage of the fused capacitor is higher than the average of the voltages of the pre-fused capacitors.

In this toy model, we have assumed that the inner and outer radii of the mitochondrial inner membrane are spheres, i.e. we have ignored cristae. If instead of assuming $A_i = 4\pi r_i^2$ we assume $A_i \propto r_i^x$ with $x \in (2, 3)$, then the final result given in Equation (1.14) stays the same. However, if we also assume that $V_i \propto r_i^y$ with $y \in (2, 3)$ then Equation (1.14) changes into

$$V_3 = (V_1 + V_2) 2^{-2/y} < 0.63 \cdot (V_1 + V_2) \quad \text{for} \quad y \in (2, 3) \tag{1.15}$$

The amount by which the voltage V_3 exceeds the average of $V_1 + V_2$ decreases as y decreases from 3 to 2, with $V_3 = \frac{1}{2}(V_1 + V_2)$ if $y = 2$. This means that in mitochondria, because of the membrane invaginations, the fused potential will be closer to the average than predicted by this simple toy model (i.e. if $V_3 = x(V_1 + V_2)$ then x will be lower than 0.63, but still potentially higher than 0.5).

1.5 Energy output by calcium stimulation as function of the number of fused mitochondria

We will discuss a very simple model that describes how the total 'energy output' of a group of mitochondria depends on the number of fused mitochondria connected to a calcium source. This simple model explains the shape of the curve in figure 4B of section 3.4.

We assume that all mitochondria that are not exposed to a calcium source have a basal $[Ca^{2+}]_{\text{matrix}}$ given by c_b. If calcium is emitted from a source (for example, a calcium channel in the ER), a mitochondrion adjacent to the source will experience an increase in $[Ca^{2+}]_{\text{matrix}}$ of c_{sig} so that its concentration now becomes $c_b + c_{sig}$, whereas mitochondria far from calcium channels keep their basal concentration.

We use a simple approximation where the notional energy output of a mitochondrion is sigmoidally dependent on $[Ca^{2+}]_{\text{matrix}}$, the matrix concentration of calcium [9]. By using the generic term energetic output we are avoiding committing to a particular physiological variable but simply seek to demonstrate the principle at play. Specifically, we denote by $s(c)$ a sigmoidal function giving the energetic output of a mitochondrion with internal calcium concentration c. The total output of the mitochondrial system, E, is then defined as

$$E = \sum_i s(c_i) \tag{1.16}$$

where i indexes mitochondria, and c_i is the calcium concentration in the ith mitochondrion.

The effect that fusion has on energy output can now be modelled. If there are N mitochondria in total, with one mitochondrion near a calcium channel (this number can easily be generalized), and n mitochondria fused to this mitochondrion, then total output is given by

$$E = (N - n) \cdot s(c_b) + n \cdot s(c_b + c_{sig}/n) \tag{1.17}$$

where the first term describes the output generated by the $(N-n)$ isolated mitochondria, and the second term gives the output of the n fused mitochondria.

In figure 4B of section 3.4 it is shown how E depends on n. To generate this figure we chose c_{sig} to be two orders of magnitude larger than c_b (as has been observed in neurons [10]) and we chose an arbitrary sigmoid function. The details of the shape of the curve depend on the form of the sigmoid used, but the general behaviour shown in the figure (E increases first and decreases again later) will remain the same. With this simple model we show that, inspired by the sigmoidal dependence of TCA cycle enzyme activity on calcium, there exists a number of fused mitochondria that gives rise to maximal energy output. The quantitative details of this result will depend on the exact sigmoidal form of enzyme activity and on c_b and c_{sig}.

1.6 Mechanisms of calcium uptake and amount of mitochondria-ER contact

Here we provide some additional information that serves as criticism of the calcium hypothesis discussed in section 3.4 of the main text. Controversy exists over kinetics of mitochondrial calcium uptake and the mechanisms involved. Besides the uniporter, other mechanisms like the Rapid Mode of uptake (RaM) and ryanodine sensitive transporters have been identified [11]. Ryanodine sensitive receptors located on the inner mitochondrial membrane become active around $[Ca^{2+}]_{cyt} \sim 100$ nM [12], whereas the mitochondrial uniporter is thought to have a low affinity for calcium with a dissociation constant around 10 μM [13]. We note, however, that the activity of the uniporter is itself Ca^{2+} dependent and the details of mitochondrial calcium influx (reviewed in [14]) are not fully understood. If uptake through ryanodine sensitive transporters is significant, then calcium uptake is not limited to mitochondria close to high $[Ca^{2+}]_{cyt}$ domains.

It is not certain what percentage of the total mitochondrial network has close contacts with the ER. There are studies suggesting this concerns ~5-20% of the total mitochondrial surface [15], and others observe that 90% of fission events occurred at sites of ER-mitochondria contact [16]. Mitochondria in the perinuclear region seem to have more ER-associations than those in the peripheral region [17]. It has been suggested that the amount of contact between mitochondria and the ER changes over time [18].

1.7 Increasing mitochondrial size buffers fluctuations in membrane potential

1.7.1 Change in membrane potential upon changes in permeability of the inner mitochondrial membrane

Here we provide arguments why, for larger mitochondria, fluctuations in membrane potential are of smaller magnitude as is stated in section 3.5 of the main text. We will use a physical model to consider how a perturbation that increases the flux of some charged chemical species into the mitochondrion (motivated by, for example, transient opening of the permeability transition pore, or proton leak), affects the membrane potential of mitochondria of different size.

The flux per unit area j_X of some chemical species X with charge z through a membrane (here the mitochondrial inner membrane) of width L is given by the Goldman equation

$$j_X = \frac{\beta \Delta\psi D_X z}{L}\left(\frac{[X]_{out} - [X]_{in} e^{\beta z \Delta\psi}}{1 - e^{\beta z \Delta\psi}}\right), \tag{1.18}$$

where $\beta = \frac{F}{RT}$, F is the Faraday constant, T the temperature, R the gas constant, $\Delta\psi$ the membrane potential, D_X the diffusion coefficient for chemical species X and $[X]_{in}$, $[X]_{out}$ are the concentrations of species X on either side of the membrane. Written in this way, the flux j_X is the net flux into the mitochondrion through a piece of unit surface area. Assuming a spherical mitochondrion with area $A = 4\pi r^2$ the total flux of X through the membrane is given by

$$J_X = \oint_A j_X dA = 4\pi r^2 \alpha \Delta\psi z \left(\frac{[X]_{out} - [X]_{in} e^{\beta z \Delta\psi}}{1 - e^{\beta z \Delta\psi}}\right), \tag{1.19}$$

where $\alpha = D_X \beta / L$ is a measure of the permeability of the membrane.

We aim to explore how robust the membrane potential is with respect to perturbations including, for example, opening of the mitochondrial permeability transition pore or proton leak. In equilibrium, the net flux of species is zero. Setting equation (1.19) to zero we can solve for $\Delta\psi$ to obtain

$$(\Delta\psi)_{equilibrium} = \frac{1}{\beta z} \ln \frac{[X]_{out}}{[X]_{in}} \tag{1.20}$$

We consider small perturbations to this equilibrium state, resulting from changes in the internal concentration of a charged species $[X]_{in}$. For a small transient perturbation, we have:

$$\frac{d\Delta\psi}{dt} = \frac{-1}{\beta z[X]_{in}} \frac{d[X]_{in}}{dt}, \tag{1.21}$$

where we have assumed that $[X]_{out}$, the cytoplasmic concentration of X, is assumed to stay constant due to its large volume compared to the mitochondrion.

Changes in $[X]_{in}$ are caused by net flux of X into the mitochondrion. Not all of these ions flowing in will increase $[X]_{in}$ as some of them may bind to proteins or ionic species inside the mitochondrion. This effect can be accounted for by a buffering factor, γ, which results in

$$\frac{d[X]_{in}}{dt} = \gamma \frac{J_X}{\frac{4}{3}\pi r^3}, \tag{1.22}$$

where we have related the absolute flux J_X to the change of concentration $[X]_{in}$ by normalizing by the volume of a (spherical) mitochondrion. The perturbation by which this influx is manifest is considered to cause an increase in permeability of the membrane, which we represent by scaling the permeability constant $\alpha \to \alpha' > \alpha$. Using equation (1.19) we obtain

$$\frac{d[X]_{in}}{dt} = \gamma\alpha'\Delta\psi z \left(\frac{[X]_{out} - [X]_{in} e^{\beta z \Delta\psi}}{1 - e^{\beta z \Delta\psi}} \right) \frac{3}{r} \tag{1.23}$$

meaning that

$$\frac{d\Delta\psi}{dt} = \frac{-\gamma\alpha'\Delta\psi}{\beta[X]_{in}} \left(\frac{[X]_{out} - [X]_{in} e^{\beta z \Delta\psi}}{1 - e^{\beta z \Delta\psi}} \right) \frac{3}{r} \tag{1.24}$$

This means that for large r (large mitochondria) fluctuations of $\Delta\psi$ will be of smaller magnitude, even if the perturbation in a larger mitochondrion happens over a larger surface area because the internal pool of ions is larger still ($r^3 > r^2$).

Treating mitochondria as spheres, we have neglected the invaginated structure of the inner membrane. Including these invaginations changes the r^{-1} dependence of $\Delta\psi$ to r^y with $y \in (-1, 0)$. Because, in spherical mitochondria (even when assuming membrane invaginations), volume will always scale with a larger power of r than surface area, fluctuations in $\Delta\psi$ will always be less in larger mitochondria. Fusing mitochondria into large networks thus protects them from perturbations.

1.7.2 Ising-type model for mitochondrial robustness

Alternative arguments independent of volume and surface area scaling can be given supporting the hypothesis discussed in section 3.5. We will briefly discuss a picture based on the Ising model, an often-used class of physical models for collective dynamics. In the Ising model, a set of elements (originally spin domains) exist, with each entity i having a state s_i, which may be -1 or 1. In a simple incarnation of the Ising model, the overall energy of the system is given by $E = \sum_i \sum_{j \geq i} J_{ij} s_i s_j$, summing over all individual elements and pairs of elements in the system, and J_{ij} is a matrix determining any energetic couplings (interactions) between elements i and j. The dynamics of the system can then be simulated by applying random perturbations to the states s and considering the change in energy that a given perturbation provokes.

If coupling is strong in a system (many off-diagonal elements of J_{ij} are of large magnitude), the effect of changing the state of one element depends strongly on the states of the elements with which it interacts. Consider an interaction matrix structured such that it is beneficial for interacting groups of elements to be in the same state (corresponding to a ferromagnetic regime, if elements represent spin domains). Strong coupling between elements will then disfavour events whereby one element changes to a state different to the state shared by its neighbours (for example, an element surrounded by elements of state 1 changing from 1 to -1). By contrast, if coupling between elements is weak, the energetic effect of such a change is localised, and the disfavouring of an isolated element changing state will be less pronounced.

We now draw an analogy to the mitochondrial system. Consider representing individual mitochondria as elements within the Ising model described above. Elements interact (with a non-zero J_{ij} term) if the corresponding mitochondria are fused; elements corresponding to isolated mitochondria do not interact with any others. We will consider the states 1 and -1 to be heuristic markers of 'functionality' and 'dysfunctionality' respectively, but it is not difficult to picture more physical interpretations, involving, for example, different states of matrix alkalinisation or membrane potential. Then, for an isolated mitochondrion to go from a functional to dysfunctional state, a change '$(1) \to (-1)$' has to occur. If several mitochondria are fused, the

required change is now placed in a coupled context e.g. '$(1)(1)(1) \to (1)(-1)(1)$'. If interactions favour similar states in coupled elements (a ferromagnetic picture), the latter change will be less favoured and will occur with lower frequency. Such ferromagnet-like interaction structure is intuitively the case, for example, when consider the concentrations of chemical species within a contiguous fused matrix, or membrane potential across a continuous membrane, where perturbations are expected to equalise across the continuous domains involved through chemical or electochemical relaxation. If, for example, a mitochondrion in a fused chain experiences a sudden change in membrane permeability, ions from neighbouring mitochondria will buffer the resulting change in $\Delta\psi$.

A chain of n fused mitochondria is then represented by a sequence of n digits and now a mitochondrion chosen randomly from this chain is likely to have two neighbours that influence its state. Glauber dynamics [19] are useful for developing intuition about such a system. In short chains, randomly chosen elements have a higher probability of having fewer neighbours, and greater variability in their individual states is therefore supported. In longer chains, strings of identical digits are more likely to occur because of the tendency of elements to take the same value as their neighbours. This, combined with the observation that mitochondria with higher $\Delta\psi$ have a higher fusion probability (in our picture, elements that are fused to a chain are more likely to be in state 1 than -1) results in an all-1 state being more favoured in longer chains than shorter chains.

Of course, this picture is only of use as a notably coarse-grained model for collective dynamics of fused mitochondria. Further progress could be made by consider continuous-state enhancements of the picture above (allowing element states to take a broader spectrum of values than 1 and -1), as required to draw a stronger quantitative connection to the behaviour of continuously-varying properties like chemical concentrations or membrane potential. With a more realistic representation of mitochondrial 'state', specific estimates of appropriate interaction terms J_{ij} could be explored.

2 Additional hypotheses

2.1 Fusion allows the possibility of creating a genetic reservoir

Summary. If mitochondria are fused they are protected from mitophagy [20, 21] which protects mtDNA mutations from being degraded, allowing the creation of a 'genetic reservoir' of mtDNA mutations. Having a reservoir of different mutations in a population can be beneficial as it allows for adaptation to the environment, increasing the 'evolvability' of the population [22]. This idea is also briefly mentioned in [23].

Note that in Section 3.1 we argued that selective fusion improves quality control, especially in the case that mitophagy is not selective. This hypothesis may require less selective fusion to permit a range of mtDNA variants to co-exist within the mitochondrial network.

Experimental support. Mitochondrial 'microheteroplasmy' [24], the universal presence of low levels of heteroplasmy, has been observed in human cells [25], supporting the idea that a pool of genetic variation is present in the mitochondrial population.

Limitations/Critique. (i) It is not immediately clear how important mitochondrial meso- or hyperfusion are in creating a genetic reservoir because the cell can fine-tune the level of quality control by changing the amount of mitochondrial biogenesis or the rate of mitophagy. (ii) We believe that even during hyperfusion quality control is present in the background ($\lambda_{fis} \neq 0$) which may reduce the size of the reservoir created. A form of static hyperfusion where mitochondria are permanently locked together would likely be physiologically damaging, as studies on hFis1 or Drp1 knockouts have suggested [26, 27, 28, 29]. (iii) This hypothesis states that fusion prevents mtDNA mutations from being lost because if the mitochondrion carrying the mutation had been fragmented, it would have been degraded. However, if a mutation causes damage to a mitochondrion, then that mitochondrion may not even be able to fuse because only mitochondria with sufficiently high $\Delta\psi$ are capable of doing so [26]. Mutations that are not harmful may indeed survive longer if they reside inside a fused network, but they could have survived without a network too, especially if the mutation gives them proliferative advantage. (iv) The link between genotype and phenotype in mitochondria is leaky [30], meaning that it may be hard for mitochondrial quality control mechanisms to select for mutated mtDNA and even without increased fusion mtDNA mutations can survive.

Experimental tests. Start with a population of wild-type heteroplasmic cells (where one type of mtDNA is pathological) and then induce mitochondrial network forming in half of them (by either decreasing fission or increasing fusion). In both populations, the relative frequencies of the two types of mtDNA can be determined. One can measure these frequencies again at a later time and observe whether more pathological mtDNAs have survived in the cells in which mitochondria were more fused.

2.2 Fusion increases ATP synthesis; hypotheses (i), (ii) and (iii)

Three hypotheses that complement section 3.3 of the main text are briefly discussed here, though in less detail than the hypotheses discussed in the main text.

2.2.1 Hypothesis (i) Higher [ATP] is caused by fusion-induced changes in inner membrane shape

Experimental support. Upon fusion, the activity of OPA1 can change cristae morphology [31, 32, 33] which may cause changes in respiratory capacity. Because ATP synthase dimers are mainly located to the crista membrane [34, 35], changes in cristae shape can alter the number of ATPase dimers embedded within the membrane [35].

Various experimental studies show support for the idea that fusion induced reshaping of the crista membrane increases respiratory capacity [36, 21]. ATP synthase dimers may be able to curve the inner mitochondrial membrane, possibly leading to the formation of new cristae [37]. Indeed, not only the ratio of ATP synthase dimers to monomers was increased during starvation, also the number of cristae per unit mitochondrial surface area was increased [21]. Another way in which changes in shape of the inner membrane can modulate energy output is by inducing changes in proton densities. Membranes with high curvature can accommodate a higher proton density than membrane with low curvature [38].

Limitations/Critique. It is not clear why fusion should be required to allow for changes in inner membrane shape; presumably a fragmented mitochondrion can do this on its own. Indeed, it has recently been found that cristae regulation of OPA1 is independent of its role in fusion [39]. It is interesting, however, that the protein involved in inner membrane fusion, OPA1, is also responsible for reshaping the cristae structure. It could be that there is a reason these two processes are linked, perhaps fusion provides more space (and more lipids) to change the cristae folding.

Experimental tests. Measure the number of cristae and ATP synthase dimers in fused filaments of mitochondria with different lengths to look for a correlation between the size of the filaments and the number of cristae and ATP dimers. Similar experiments have been done before in which both cristae- and dimer number were observed to increase upon starvation induced hyperfusion [21]. Starvation, however, will affect the cell and it may be that the increases in cristae and dimer number are not a direct consequence of increased fusion but have other origins. Because mitochondrial length is often heterogeneous in wild-type cells, one can use these cells to perform measurements.

2.2.2 Hypothesis (ii) Higher [ATP] is caused by fusion-induced decreases in proton leak

Proton leakage across the inner mitochondrial membrane leads to decreased efficiency of the electron transport chain machinery [40]. Fusion could lead to a decrease in the amount of proton leak, meaning that ATP production would become more efficient in a fused state (more ATP produced per substrate reduced).

Experimental support. This idea is supported by the observation that fragmentation of mitochondria in brown adipose tissue promotes a shift from using nutrient oxidation for ATP synthesis, to using it for heat generation (which is caused by increased proton leak) [41]. In this study they find that fragmentation is required to induce this increased proton leak in hormonally stimulated brown adipocytes [41]. Mitochondrial dynamics might thus change the efficiency of ATP production, with efficiency being high if mitochondria are fused and low in a fragmented state. The mechanism by which leak was observed to decrease after fusion was related to the protein Uncoupling Protein 1 (Ucp1) [41], which is specific to brown adipocytes. Other tissues do contain isoforms of Ucp1, called Ucp2, Ucp3, Ucp4 and Ucp5.

Limitations/Critique. This hypothesis is based on measurements of leak in brown adipose tissue but it is not clear that the results hold for other tissues as well. Brown adipose tissue is specialized in heat generation and proton leak may play a less important role in other tissues. Morever, the mechanism causing the changes in leak involved the protein Uncoupler Protein 1 (Ucp1) [41] which is specific to brown adipocytes and therefore this mechanism may not exist in other tissues.

Experimental tests. Measure whether there exists a correlation between the amount of proton leak and the length of a mitochondrial filament in cells other than brown adipocytes. To avoid side effects from overexpressing or downregulating specific proteins, one can use the natural heterogeneity of mitochondrial length in a cell.

The kinetics of the proton leak can be determined indirectly, by measuring the oxygen consumption of mitochondria under non-phosphorylating conditions (plus oligomycin) as a function of the proton electrochemical gradient.

2.2.3 Hypothesis (iii) Higher [ATP] is caused by fusion-induced decreases in mitochondrial degradation

Mitochondria that are part of a large network are spared from mitophagy and it has been suggested that hyperfusion is a mechanism to protect mitochondria from degradation [21, 20]. The cause of less degradation during hyperfusion can simply be that only mitochondria under a certain threshold size can be captured by an autophagosomal membrane. Degradation of mitochondria, however, happens for a reason and stopping this degradation can have negative consequences.

Experimental support. Inhibiting mitophagy causes reductions in ATP levels in the cell and more ROS production, most likely because damaged mitochondria are not removed anymore [42]. We also note that the idea of less degradation during hyperfusion contrasts our observation that increased fusion can increase mitochondrial selection even in the absence of selective mitophagy.

Limitations/Critique. This hypothesis relies on an increase in mitochondrial mass as a result of a decrease in mitochondrial degradation. Whether this is true, however, is not clear. The cell may have control over total mitochondrial mass (energy production is crucial for the cell and one may expect it to be highly controlled) and reduce mitochondrial biogenesis in response to decreases in mitophagy. There could of course be other reasons for wanting to reduce rates of mitophagy that we are yet unaware of.

Experimental tests. Measure mitochondrial mass and the amount of mitophagy in a fragmented state and a hyperfused state to see whether decreases in mitophagy levels (which are known to occur in hyperfused states) lead to increases in mitochondrial mass. It is also interesting to measure whether the cell is homeostatic with respect to mitochondrial mass and responds to decreased mitophagy by decreasing mitochondrial biogenesis.

2.3 Fusion creates distributional 'power cables'

Summary. Fused mitochondria facilitate the distribution of respiratory capacity through the cell. If mitochondria form long extended networks, then the electrochemical gradient may equilibrate along the whole filament (by movement of protons along mitochondria), making it possible for a mitochondrion at one end of the chain to use the electrochemical potential generated by a mitochondrion at another end of the chain to create ATP. Oxygen and nutrient levels will be higher at the cell's periphery and therefore mitochondria in the middle of the cell can take advantage of the electrochemical gradient produced by peripheral mitochondria (because oxygen is required to create the gradient). Mitochondrial networks may thus be used as 'power cables' to enable power transmission across the cell [43, 44, 45, 46]. An illustration is given in Figure 3C in the main text.

The cable mechanism suggests several cellular advantages. 3D diffusion of ATP or oxygen through the cytoplasm can be replaced by 1D proton movement along mitochondrial filaments; the speed of energy transmission is therefore likely to increase due to the reduced dimensionality and increasingly directed nature of the motion. Another advantage is that cabling may reduce damage by reactive oxygen species (ROS) in the core of the cell where nuclear DNA is present. This is because ROS is generated at sites where the ETC is active and the power cabling hypothesis predicts that this is mainly at the cell periphery.

Experimental support. When ATP is used more in certain parts of the cell, mitochondria tend to cluster around these parts, suggesting that ATP supply from distal mitochondria to these regions is not enough [47]. Several tissues have very limited mitochondrial movement (e.g. heart cells [48]); cabling could provide a way to ensure sufficient ATP supply to these regions.

Coarse-grained quantitative model. To make progress exploring the implications of the cable hypothesis for ATP production, we first estimate the speed gain obtained by using cables by comparing the diffusion time of oxygen through the cytoplasm with that of H^+ along mitochondrial membranes. Performing this calculation with values measured in biological experiments suggests that cables facilitate an order-of-magnitude increase in ATP production (see below), thus providing a quantitative suggestion that mitochondrial power cables are beneficial to the cell.

Further modelling. The flow of electricity in a leaky cable (a long cylindrical piece of membrane) is described by the cable equation [49]. The cable equation is used to study electrical flow in neurons but may be applied to a chain of electrically continuous mitochondria as well, providing more fine-grained physical insight into the electrochemical dynamics of mitochondrial power cables.

Limitations/Critique. The real speedup of ATP synthesis rate obtained by using cables instead of diffusion of oxygen, is likely to be smaller than our quantitative estimate taking into account that silent fission events (measured to occur with a probability around 50% over five minutes in a filament not longer than 4μm [50]) will slow down the diffusing particle. The complex that actually uses oxygen in the respiratory chain (complex IV) has a reaction capacity which normally exceeds the maximal rate of the respiratory chain and can operate at low oxygen tension [51], which weakens the statement that oxygen supply is a rate limiting step in respiration. The cabling hypothesis may represent a reason for mitochondrial fusion in skeletal muscle cells, as these are

highly dependent on oxygen supply and have high ATP demands. It is less clear, however, how meso- and hyperfusion can be explained in other tissues less dependent on oxygen and with a lower ATP demand. The hypothesis also has difficulties explaining the high frequency of fission and fusion rate. Finally, H_2O_2, a source for ROS damage, is able to diffuse freely across several cell lengths and can thus induce damage far from the site of ROS generation [52], questioning the ability of cabling to reduce ROS damage in the core of the cell where nuclear DNA is present.

Experimental tests. The power cabling hypothesis makes several predictions that can be tested experimentally: in stimulated red muscle fibre i) the ETC is more active in the periphery of the cell than in the cell core, ii) there exists an oxygen gradient between the periphery of the cell and the cell core.

2.3.1 Order of magnitude increase in energy production using mitochondrial power cabling

We here provide an explanation of the claim made above that cabling facilitates an order-of-magnitude increase in ATP production. This quantitative argument proceeds by considering the ATP generation in a central region of the cell where oxygen concentration is low. We will compare two cases. Firstly, a 'null' case with no cabling, where oxygen must diffuse from a peripheral region of the cell to act as an electron acceptor for ETC complexes in this central region. Secondly, a 'cabling' case, where proton pumping is performed by ETC complexes in the oxygen-rich peripheral region, then protons are 'cabled' to central, low-oxygen region to facilitate ATP production.

We will discuss diffusion of oxygen in skeletal muscle cells because the cable hypothesis was developed studying these cells. We will use a diffusion coefficient of $D_{O_2} = (2.7 \pm 0.3) \times 10^{-7}$ cm^2/s [53, 54] for oxygen diffusing through the cytoplasm. For protons we set $D_{H^+} = (5.7 \pm 0.7) \times 10^{-5}$ cm^2/s [55]. The proton diffusion coefficient was calculated assuming 2D diffusion so in our estimation we should treat it as such [55] (the calculated value of D_{H^+} would be different if 1D diffusion was assumed, so the diffusion constant obtained in [55] is specific for 2D diffusion). We argued before that proton diffusion along mitochondrial membranes can be described as a 1D diffusion process which increases the diffusion speed. We are merely providing an order of magnitude estimation here so for our purposes we can ignore dimensionality ambiguities. Assuming random diffusion (no bias) then the ratio of the time for oxygen to diffuse across a distance x, t_{O_2}, to that of protons, t_H^+, is given by

$$\frac{t_{O_2}}{t_H^+} \approx \frac{D_H^+}{D_{O_2}} \approx 214 \pm 36. \tag{2.1}$$

Suppose the number of protons necessary for the production of one ATP molecule is n ($n \approx 3$). The net reaction of complexes I, III and IV of the respiratory chain is

$$2NADH + 22H^+_{matrix} + O_2 \quad \rightarrow \quad 2NAD^+ + 20H^+_{IMS} + 2H_2O, \tag{2.2}$$

hence, for every oxygen molecule used, 20 protons are pumped into the intermembrane space.

We now compare ATP production in the case where oxygen concentration is limiting. We assume that the flux of n protons down the electrochemical gradient and through complex V is required to create one ATP molecule. In the case of a limiting oxygen concentration, the presence of one oxygen molecule thus facilitates the production of about $20/n$ ATP molecules, or 20 times more than are produced through the presence of one proton. The rate at which oxygen is supplied to a mitochondrion can thus be 20 times less than the rate at which protons are supplied while still resulting in the same rate of ATP synthesis. Combining this with Equation (2.1), we estimate that using proton diffusion along mitochondrial membranes to generate ATP where oxygen diffusion is limited, the rate of ATP production will be about an order of magnitude larger.

2.4 Meso- and hyperfusion have no function

Summary. It is possible that mitochondrial meso- and hyperfusion bestows no additional advantage to the cell. Changes in the balance between fusion and fission events can arise naturally as a consequence of changes in activities of mitochondrial fission and fusion proteins and other aspects of cell physiology, and large-scale fusion may represent an epiphenomenon, emerging from variations in these features with distinct functional consequences. Fusion may also be a side effect of mitochondrial clustering in regions of high ATP demand. Localized demand would then cause fusion but the fusion itself would impart no benefit (of course clustering might be caused by localized demand but fusion might give an energetic or quality control advantage as discussed).

Experimental support. Mammalian mitochondria are connected to microtubules via motor proteins that mediate their (ATP-dependent) transport, and, because of this connectivity, long strings of mitochondria can form [56]. These 'long bundles' of mitochondria have been observed in cardiomyocytes [48]. This manner of

alignment can facilitate connected filaments to arise when changes occur in the activities of mitochondrial shaping proteins because the mitochondria are already lying end-to-end, ready to form a continuous filament. Changes in the microtubule network may also alter mitochondrial morphology.

The main fusion and fission proteins, OPA1, MFN1,2 and Drp1, all have other functions in the cell and Drp1 can undergo numerous post-translational modifications. A list of these additional functions and post-translational modifications is given in Tables 4 and 5. Also OPA1 undergoes modifications and can be cleaved to its shorter isoforms by various proteins [57, 58, 59, 60, 61, 62].

We stress that the fact that many different proteins and post-translational modifications have effects on mitochondrial morphology can lead to two entirely different conclusions: i) mitochondrial dynamics has a highly important function and needs to be controlled by various pathways, and ii) mitochondrial dynamics is an epiphenomenon and changes in morphology occur because of natural fluctuations in the activities of the mitochondrial shaping proteins.

Experimental tests. We do not propose approaches to model or experimentally test this case; instead suggesting that it is regarded as the 'null hypothesis' against which other alternative hypotheses for mitochondrial network formation may be compared.

3 Tables

Condition under which extended fusion is observed	Cell type	Cause of extended fusion	Ref.
G1-S or G1 phase of the cell cycle	Normal rat kidney cells (G1-S) and human osteosarcoma cells (G1)	Not reported	[27, 63]
Various kinds of stress including starvation, oxidative stress, UV irradiation and cycloheximide	Various cell types including MEFs, mouse primary fibroblasts, hepatocytes, human HeLa and Cos-7 cells and HEK293T cells	In the case of starvation, decreased Drp1 Ser-616 and increased Drp1 Ser-637 phosphorylation was observed [20, 21]. The stress induced hyperfusion studied by [36] was suggested to be caused by increased fusion rather than decreased fission	[36, 64, 65, 21, 20]
In long-living individuals (100.67 $\pm$ 2.88 years) mitochondria formed more fused, elongated structures than in young (27.83 $\pm$ 3.97 years) or old (75.67 $\pm$ 10.86 years) individuals	Dermal fibroblasts	The cause was suggested to be mainly decreased fission rather than increased fusion	[66]
Mitochondrial length increased during aging, senescent cells shows long and interconnected mitochondria	HUVEC cells	A reduced expression of hFis1 and Drp1 was observed	[67]
Prolonged incubation with 20 nM MitoQ, an antioxidant	HeLa cells and normal human fibroblasts	Suggested to be decreased fission	[68]
Cells grown in glucose rather than galactose media	HeLa cells	Not reported	[69]

Table 1: **Occurrences and causes of hyperfusion in wild-type cells.** Here we list conditions under which wild-type cells show highly fused mitochondrial structures. The cause of the fused morphology (increased fusion, decreased fission or both) is reported if known.

Normal morphological state	Cell type	Ref.
- 1 day old cells show mainly tubular or intermediate (both fragmented and elongated) morphologies<br>- 5 days old cells show an intermediate morphology<br>- 11 days old cells show intermediate or very fragmented mitochondria<br>- 16 days old cells show mostly very fragmented mitochondria	C. elegans body wall muscle	[70]
Typical cells show mitochondria dispersed throughout the cytosol and their shape seems to be round, ellipsoid-, or sausage-like	human Cos-7 cells	[71]
Wild-type cells typically show a tubular network structure	MEFs	[72]
Under standard culturing conditions, 40% of the cells had mostly tubular mitochondria, 50% mostly fragmented mitochondria and 10% of the cells had an intermediate state (in which both fragmented and branched mitochondria coexisted in roughly equal amounts)	human osteosarcoma cells	[63]
Mitochondria in wild-type cells grown in media containing galactose appear interconnected. Shifting cells from aerobic to anaerobic growth did not affect mitochondrial structure (at least not within 7 hours)	yeast *S. cerevisiae*	[73]
Both glucose and glycerol-grown cells show a single large mitochondrial component with few fragmented smaller ones. The large mitochondrial component was about four times more branched in glycerol grown cells, and mitochondrial volume was three times larger in glycerol grown cells.	yeast *S. cerevisiae*	[74]
- Cells grown in media with 2% glucose showed an elongated mitochondrial structure<br>- in media with 0.5% glucose mitochondria appeared shorter<br>- in media with 4% glucose mitochondria had few connections and branches	yeast *S. cerevisiae*	[75]
- Cells grown in glucose cultures show elongated and branched mitochondria<br>- Cells grown in ethanol have smaller/shorter mitochondria compared to those grown in glucose cultures (total mitochondrial volume was the same)<br>- Cells in aerobic glucose-limited chemostat cultures have many short mitochondria<br>- Cells in anaerobic fermentative chemostat cultures have elongated mitochondria	yeast *S. cerevisiae*	[76]

Table 2: **Typical mitochondrial morphology in a variety of cells.** To obtain a better idea of the 'normal' morphological state of mitochondria, we give an overview of observed 'typical' mitochondrial distributions. One has to keep in mind that the morphological state depends on cell function and cell cycle. Many studies investigate mitochondrial morphology by staining with TMRE, JC-1 (both of which are dyes for mitochondrial membrane potential) or matrix targeted GFP (Green Fluorescent Protein). However, as was stated in [50], 'the non-fragmented shape of the web as revealed by TMRE alone does not reliably predict the boundaries of a network'. It is therefore hard to know exactly how fused mitochondria actually were in studies that do not check for continuity with photoactivatable GFP.

Species of interest	Diffusion coefficient ($\mu m^2/s$)	Ref.
Complex V:<br>(mobile fraction)<br>(less mobile fraction)<br>(immobile fraction)	 0.082 ± 0.002 (s.e.m.)<br>0.019 ± 0.001 (s.e.m.)<br>0.006 ± 0.001 (s.d.)	[77]
Complex II:<br>(mobile fraction)<br>(less mobile fraction)<br>(immobile fraction, ($\leq 15\%$))	 0.140 ± 0.005 (s.e.m.)<br>0.039 ± 0.002 (s.e.m.)<br>0.004	[77]
Matrix targeted GFP	20-30	[78]
Matrix targeted EYFP:<br>In stationary mitochondria<br>In moving mitochondria	 22 ± 2.1 (s.d., N = 12)<br>18 ± 4.7 (s.d., N = 46)	[79]
Mobile fraction of TFAM labelled mtDNA	0.05 ± 0.01	[30]
Mobile fraction ($93 \pm 6\%$) of outer membrane protein TOM7	0.7-1	[80]
Mobile fraction ($93 \pm 5\%$) of outer membrane protein hFis1	0.4-1	[80]
Outer membrane protein Tom40	0.5	[81]

Table 3: **Diffusion constants of several mitochondrial proteins.**

Modifier	Modification effect on Drp1	Ref.
Protein Kinase A (PKA)	Phosphorylation of Drp1 at Ser-637 which prevents Drp1 from tethering to mitochondria. Drp1 remains retained in the cytosol instead.	[82]
CaM kinase Iα, ROCK1	Phosphorylation of Drp1 at Ser-637 which caused increased mitochondrial fission	[83, 84]
Cdk1, Cdk5, PKCδ, Erk1/2	Phosphorylation of Drp1 at Ser-616 which activates Drp1	[85, 86, 87, 88]
Calcineurin	Dephosphorylation of Drp1 at Ser-637 making Drp1 capable of translocating to mitochondria	[89]
MAPL, SUMO1,2,3	Sumoylation of Drp1 which affects it GTPase cycle	[90, 91]
O-GlcNAc-transferase and N-acetyl-glucosaminidase	O-linked-N-acetyl-glucosamine glycosylation of Drp1 at threonine 585 or 586. This decreases phosphorylation at Ser-637 and results in increased translocation of Drp1 to mitochondria	[92]
Bax and Bak	Promotion of sumoylation of Drp1 during apoptosis	[93]
MITOL/MARCH5	Ubiquitination of both hFis1 and Drp1	[94, 95]

Table 4: **Overview of various post-translational modifications of the GTPase Drp1 which mediates mitochondrial fission.**

Protein name	Processes other than fusion and fission in which the protein is involved	Role of the protein	Ref.
OPA1	- Regulation of cristae morphology	- OPA1 oligomers may wrap around crista junctions to keep them tight	[32, 31]
	- Apoptosis	- By tightening crista junctions, cytochrome c is kept inside the cristae instead of diffusing into the cytosol and inducing apoptosis	[32, 96, 31]
	- Depolarization events	- OPA1 causes reversible depolarization events after matrix contractions	[97, 98]
MFN2	- Mitochondria-ER interactions and calcium homeostasis	- MFN2 tethers mitochondria to the ER	[99]
	- Oxidative phosphorylation gene expression	- Independently of its role in fusion, MFN2 regulates expression of OXPHOS genes	[100]
	- Apoptosis	- A MFN2 mutant has been observed to increase cytochrome c release following DNA damage and ROS damage, suggesting that MFN2, just like OPA1, works to keep cytochrome c inside the cristae	[101]
MFN1	Apoptosis	MFN1 may regulate activation of the pro-apoptotic protein Bax	[102]
Drp1	Apoptosis	Drp1 is transported to mitochondria upon apoptosis induction. Inhibition of Drp1 can prevent apoptosis	[103]

Table 5: **Functions of the mitochondrial fusion and fission proteins other than inducing mitochondrial fusion or fission.**

References

[1] **Phillips R, Milo R**. 2009. A feeling for the numbers in biology. *Proc Natl Acad Sci USA* **106**: 21465–21471.

[2] **Moran U, Phillips R, Milo R**. 2010. SnapShot: key numbers in biology. *Cell* **141**: 1262–1262.

[3] **Harrison AK, Zwanzig R**. 1985. Transport on a dynamically disordered lattice. *Phys Rev A* **32**: 1072.

[4] **Sahimi M**. 1986. Dynamic percolation and diffusion in disordered systems. *J Phys C: Solid State Phys* **19**: 1311.

[5] **Kirkpatrick S**. 1973. Percolation and conduction. *Rev Mod Phys* **45**: 574.

[6] **Junesch U, Graber P**. 1991. The rate of ATP-synthase as a function of dpH and dpsi catalyzed by the active, reduced H+-ATPase from chloroplasts. *FEBS* **294**: 275–278.

[7] **Soga N, Kinosita K, Yoshida M, Suzuki T**. 2012. Kinetic Equivalence of Transmembrane pH and Electrical Potential Differences in ATP Synthesis. *J Biol Chem* **287**: 9633–9639.

[8] **Beard DA**. 2006. A Biophysical Model of the Mitochondrial Respiratory System and Oxidative Phosphorylation. *PLoS Comput Biol* **1**: e36.

[9] **Wan B, LaNoue KF, Cheung JY, Scaduto RC**. 1989. Regulation of citric acid cycle by calcium. *J Biol Chem* **264**: 13430–9.

[10] **Ivannikov MV, Macleod GT**. June 2013. Mitochondrial free Ca levels and their effects on energy metabolism in Drosophila motor nerve terminals. *Biophys J* **104**: 23532361.

[11] **Dedkova EN, Blatter LA**. 2013. Calcium signaling in cardiac mitochondria. *J Mol Cell Cardiol* **58**: 125 – 133.

[12] **Altschafl BA, Beutner G, Sharma VK, Sheu SS,** et al. 2007. The mitochondrial ryanodine receptor in rat heart: A pharmaco-kinetic profile. *Biochim Biophys Acta* **1768**: 1784 – 1795.

[13] **Duszyski J, Kozie R, Brutkowski W, Szczepanowska J,** et al. 2006. The regulatory role of mitochondria in capacitative calcium entry. *Biochim Biophys Acta* **1757**: 380 – 387.

[14] **Ahuja M, Muallem S**. 2014. The gatekeepers of mitochondrial calcium influx: MICU1 and MICU2. *EMBO Rep* **15**: 205–206.

[15] **Rizzuto R, Pinton P, Carrington W, Fay FS,** et al. 1998. Close Contacts with the Endoplasmic Reticulum as Determinants of Mitochondrial Ca2+ Responses. *Science* **280**: 1763–1766.

[16] **Friedman JR, Lackner LL, West M, DiBenedetto JR,** et al. 2011. ER Tubules Mark Sites of Mitochondrial Division. *Science* **334**: 358–362.

[17] **Collins TJ, Bootman MD**. 2003. Mitochondria are morphologically heterogeneous within cells. *J Exp Biol* **206**: 1993–2000.

[18] **Pacher P, Sharma K, Csordás G, Zhu Y,** et al. 2008. Uncoupling of ER-mitochondrial calcium communication by transforming growth factor-. *Am J Physiol Renal Physiol* **295**: F1303–F1312.

[19] **Martinelli F**. 1999. Lectures on Glauber dynamics for discrete spin models. In *Lectures on probability theory and statistics*, Springer, 93–191.

[20] **Rambold AS, Kostelecky B, Elia N, Lippincott-Schwartz J**. 2011. Tubular network formation protects mitochondria from autophagosomal degradation during nutrient starvation. *Proc Natl Acad Sci USA* **25**: 10190 – 5.

[21] **Gomes LC, Benedetto GD, Scorrano L**. 2011. During autophagy mitochondria elongate, are spared from degradation and sustain cell viability. *Nat Cell Biol* **13**: 589.

[22] **Pigliucci M**. 2008. Is evolvability evolvable? *Nat Rev Genet* **9**: 75–82.

[23] **Rodell AB, Rasmussen LJ, Bergersen LH, Singh KK,** et al. 2013. Natural Selection of Mitochondria During Somatic Lifetime Promotes Healthy Aging. *Front Neuroenergetics* **5**.

[24] **Smigrodzki RM, Khan SM**. 2005. Mitochondrial microheteroplasmy and a theory of aging and age-related disease. *Rejuvenation Res* **8**: 172–198.

[25] **Payne BA, Wilson IJ, Yu-Wai-Man P, Coxhead J,** et al. 2013. Universal heteroplasmy of human mitochondrial DNA. *Hum Mol Genet* **22**: 384–390.

[26] **Twig G, Elorza A, Molina AJ, Mohamed H,** et al. 2008. Fission and selective fusion govern mitochondrial segregation and elimination by autophagy. *EMBO J* **27**: 433–446.

[27] **Mitra K, Wunder C, Roysam B, Lin G,** et al. 2009. A hyperfused mitochondrial state achieved at G1S regulates cyclin E buildup and entry into S phase. *Proc Natl Acad Sci USA* **106**: 11960–11965.

[28] **Parone PA, Da Cruz S, Tondera D, Mattenberger Y,** et al. 2008. Preventing mitochondrial fission impairs mitochondrial function and leads to loss of mitochondrial DNA. *PLos ONE* **3**: e3257.

[29] **Qian W, Choi S, Gibson GA, Watkins SC,** et al. 2012. Mitochondrial hyperfusion induced by loss of the fission protein Drp1 causes ATM-dependent G2/M arrest and aneuploidy through DNA replication stress. *J Cell Sci* **125**: 5745–5757.

[30] **Busch KB, Kowald A, Spelbrink JN**. 2014. Quality matters: how does mitochondrial network dynamics and quality control impact on mtDNA integrity? *Philos Trans R Soc Lond B Biol Sci* **369**: 1–12.

[31] **Cipolat S, Rudka T, Hartmann D, Costa V,** et al. 2006. Mitochondrial Rhomboid {PARL} Regulates Cytochrome c Release during Apoptosis via OPA1-Dependent Cristae Remodeling. *Cell* **126**: 163 – 175.

[32] **Frezza C, Cipolat S, de Brito OM, Micaroni M,** et al. 2006. {OPA1} Controls Apoptotic Cristae Remodeling Independently from Mitochondrial Fusion. *Cell* **126**: 177 – 189.

[33] **Olichon A, Baricault L, Gas N, Guillou E,** et al. 2003. Loss of OPA1 Perturbates the Mitochondrial Inner Membrane Structure and Integrity, Leading to Cytochrome c Release and Apoptosis. *J Biol Chem* **278**: 7743–7746.

[34] **Gilkerson RW, Selker JM, Capaldi RA**. 2003. The cristal membrane of mitochondria is the principal site of oxidative phosphorylation. *FEBS Lett* **546**: 355 – 358.

[35] **Jimenez L, Laporte D, Duvezin-Caubet S, Courtout F,** et al. 2013. Mitochondrial ATP synthases cluster as discrete domains that reorganize with the cellular demand for oxidative phosphorylation. *J Cell Sci* **127**: 719–26.

[36] **Tondera D, Grandemange S, Jourdain A, Karbowski M,** et al. 2009. SLP-2 is required for stress-induced mitochondrial hyperfusion. *EMBO J* **28**: 1589–1600.

[37] **Davies KM, Anselmi C, Wittig I, Faraldo-Gmez JD,** et al. 2012. Structure of the yeast F1Fo-ATP synthase dimer and its role in shaping the mitochondrial cristae. *Proc Natl Acad Sci USA* **109**: 13602–13607.

[38] **Strauss M, Hofhaus G, Schröder RR, Kühlbrandt W**. 2008. Dimer ribbons of ATP synthase shape the inner mitochondrial membrane. *EMBO J* **27**: 1154–1160.

[39] **Patten DA, Wong J, Khacho M, Soubannier V,** et al. 2014. OPA1-dependent cristae modulation is essential for cellular adaptation to metabolic demand. *EMBO J* **33**: 2676–2691.

[40] **Divakaruni AS, Brand MD**. 2011. The Regulation and Physiology of Mitochondrial Proton Leak. *Physiology* **26**: 192–205.

[41] **Wikstrom JD, Mahdaviani K, Liesa M, Sereda SB,** et al. 2014. Hormone-induced mitochondrial fission is utilized by brown adipocytes as an amplification pathway for energy expenditure. *EMBO J* .

[42] **Melser S, Chatelain EH, Lavie J, Mahfouf W,** et al. 2013. Rheb Regulates Mitophagy Induced by Mitochondrial Energetic Status. *Cell Metab* **17**: 719 – 730.

[43] **Bakeeva L, Chentsov Y, Skulachev V**. 1978. Mitochondrial framework (reticulum mitochondriale) in rat diaphragm muscle. *Biochim Biophys Acta* **501**: 349–369.

[44] **Skulachev VP**. 1990. Power Transmission along Biological Membranes. *J Membrane Biol* **114**: 97.

[45] **Amchenkova AA, Bakeeva LE, Chentsov YS, Skulachev VP,** et al. 1988. Coupling membranes as energy-transmitting cables. I. Filamentous mitochondria in fibroblasts and mitochondrial clusters in cardiomyocytes. *J Cell Biol* **107**: 481–495.

[46] **Skulachev VP**. 2001. Mitochondrial filaments and clusters as intracellular power-transmitting cables. *Trends Biochem Sci* **26**: 23 – 29.

[47] **Jones Dt**. 1986. Intracellular diffusion gradients of O2 and ATP. *Am J Physiol* **250**: C663–C675.

[48] **Huang X, Sun L, Ji S, Zhao T,** et al. 2013. Kissing and nanotunneling mediate intermitochondrial communication in the heart. *Proc Natl Acad Sci USA* **110**: 2846–2851.

[49] **Keener JP, Sneyd J**. 1998. *Mathematical physiology*, volume 8. Springer.

[50] **Twig G, Graf SA, Wikstrom JD, Mohamed H,** et al. 2006. Tagging and tracking individual networks within a complex mitochondrial web with photoactivatable GFP. *Am J Physiol Cell Physiol* **291**: C176–C184.

[51] **Gnaiger E, Kuznetsov A**. 2002. Mitochondrial respiration at low levels of oxygen and cytochrome c. *Biochem Soc Trans* **30**: 252–257.

[52] **Kirkinezos IG, Moraes CT**. 2001. Reactive oxygen species and mitochondrial diseases. *Seminars in Cell and Developmental Biology* **12**: 449 – 457.

[53] **Moll W**. 1968. The diffusion coefficient of myoglobin in muscle homogenate. *Pflger's Archiv fr die gesamte Physiologie des Menschen und der Tiere* **299**: 247–251.

[54] **Stroeve P**. 1982. Myoglobin-facilitated oxygen transport in heterogeneous red muscle tissue. *Annals of Biomedical Engineering* **10**: 49–70.

[55] **Zhang C, Knyazev DG, Vereshaga YA, Ippoliti E,** et al. 2012. Water at hydrophobic interfaces delays proton surface-to-bulk transfer and provides a pathway for lateral proton diffusion. *Proc Natl Acad Sci USA* **109**: 9744–9749.

[56] **Heggeness MH, Simon M, Singer SJ**. 1978. Association of mitochondria with microtubules in cultured cells. *Proc Natl Acad Sci USA* **75**: 3863–3866.

[57] **Song Z, Chen H, Fiket M, Alexander C,** et al. 2007. OPA1 processing controls mitochondrial fusion and is regulated by mRNA splicing, membrane potential, and Yme1L. *J Cell Biol* **178**: 749–755.

[58] **Guillery O, Malka F, Landes T, Guillou E,** et al. 2008. Metalloprotease-mediated OPA1 processing is modulated by the mitochondrial membrane potential. *Biol Cell* **100**: 315–325.

[59] **Mishra P, Carelli V, Manfredi G, Chan DC**. 2014. Proteolytic Cleavage of Opa1 Stimulates Mitochondrial Inner Membrane Fusion and Couples Fusion to Oxidative Phosphorylation. *Cell Metab* **19**: 630 – 641.

[60] **Baricault L, Sgui B, Gugand L, Olichon A,** et al. 2007. {OPA1} cleavage depends on decreased mitochondrial {ATP} level and bivalent metals. *Experimental Cell Research* **313**: 3800 – 3808.

[61] **Duvezin-Caubet S, Jagasia R, Wagener J, Hofmann S,** et al. 2006. Proteolytic Processing of OPA1 Links Mitochondrial Dysfunction to Alterations in Mitochondrial Morphology. *J Biol Chem* **281**: 37972–37979.

[62] **Ishihara N, Fujita Y, Oka T, Mihara K**. 2006. Regulation of mitochondrial morphology through proteolytic cleavage of OPA1. *EMBO J* **25**: 2966–2977.

[63] **Margineantu DH, Cox WG, Sundell L, Sherwood SW,** et al. 2002. Cell cycle dependent morphology changes and associated mitochondrial {DNA} redistribution in mitochondria of human cell lines. *Mitochondrion* **1**: 425 – 435.

[64] **Redpath CJ, Khalil MB, Drozdal G, Radisic M,** et al. 2013. Mitochondrial Hyperfusion during Oxidative Stress Is Coupled to a Dysregulation in Calcium Handling within a C2C12 Cell Model. *PLos ONE* **7**: e69165.

[65] **Zemirli N, Pourcelot M, Ambroise G, Hatchi E,** et al. 2014. Mitochondrial hyperfusion promotes NF-B activation via the mitochondrial E3 ligase MULAN. *FEBS J* **14**: 3095–112.

[66] **Sgarbi G, Matarrese P, Pinti M, Lanzarini C,** et al. 2014. Mitochondria hyperfusion and elevated autophagic activity are key mechanisms for cellular bioenergetic preservation in centenarians. *Aging (Albany NY)* **6**: 296.

[67] **Mai S, Klinkenberg M, Auburger G, Bereiter-Hahn J,** et al. 2010. Decreased expression of Drp1 and Fis1 mediates mitochondrial elongation in senescent cells and enhances resistance to oxidative stress through PINK1. *J Cell Sci* **123**: 917–926.

[68] **Pletjushkina O, Lyamzaev K, Popova E, Nepryakhina O,** et al. 2006. Effect of oxidative stress on dynamics of mitochondrial reticulum. *Biochim Biophys Acta* **1757**: 518 – 524, 14th European Bioenergetics Conference.

[69] **Rossignol R, Gilkerson R, Aggeler R, Yamagata K,** et al. 2004. Energy substrate modulates mitochondrial structure and oxidative capacity in cancer cells. *Cancer Res* **64**: 985–993.

[70] **Regmi SG, Rolland SG, Conradt B**. 2014. Age-dependent changes in mitochondrial morphology and volume are not predictors of lifespan. *Aging (Albany NY)* **6**: 118.

[71] **Smirnova E, Shurland DL, Ryazantsev SN, van der Bliek AM**. 1998. A Human Dynamin-related Protein Controls the Distribution of Mitochondria. *J Cell Biol* **143**: 351–358.

[72] **Chen H, Chomyn A, Chan DC**. 2005. Disruption of Fusion Results in Mitochondrial Heterogeneity and Dysfunction. *J Biol Chem* **280**: 26185–26192.

[73] **Nunnari J, Marshall WF, Straight A, Murray A,** et al. 1997. Mitochondrial transmission during mating in Saccharomyces cerevisiae is determined by mitochondrial fusion and fission and the intramitochondrial segregation of mitochondrial DNA. *Mol Biol Cell* **7**: 1233–42.

[74] **Egner A, Jakobs S, Hell SW**. 2002. Fast 100-nm resolution three-dimensional microscope reveals structural plasticity of mitochondria in live yeast. *Proc Natl Acad Sci USA* **99**: 3370–3375.

[75] **Aung-Htut MT, Lam YT, Lim YL, Rinnerthaler M,** et al. 2013. Maintenance of mitochondrial morphology by autophagy and its role in high glucose effects on chronological lifespan of Saccharomyces cerevisiae. *Oxid Med Cell Longev* **2013**.

[76] **Visser W, van Spronsen E, Nanninga N, Pronk J,** et al. 1995. Effects of growth conditions on mitochondrial morphology inSaccharomyces cerevisiae. *Antonie van Leeuwenhoek* **67**: 243–253.

[77] **Wilkens V, Kohl W, Busch K**. 2013. Restricted diffusion of OXPHOS complexes in dynamic mitochondria delays their exchange between cristae and engenders a transitory mosaic distribution. *J Cell Sci* **126**: 103–116.

[78] **Partikian A, Iveczky B, Swaminathan R, Li Y,** et al. 1998. Rapid Diffusion of Green Fluorescent Protein in the Mitochondrial Matrix. *J Cell Biol* **140**: 821–829.

[79] **Koopman WJ, Hink MA, Verkaart S, Visch HJ,** et al. 2007. Partial complex I inhibition decreases mitochondrial motility and increases matrix protein diffusion as revealed by fluorescence correlation spectroscopy. *Biochim Biophys Acta* **1767**: 940 – 947.

[80] **Sukhorukov VM, Dikov D, Busch K, Strecker V,** et al. 2010. Determination of protein mobility in mitochondrial membranes of living cells. *Biochim Biophys Acta* **1798**: 2022 – 2032.

[81] **Kuzmenko A, Tankov S, English BP, Tarassov I,** et al. 2011. Single molecule tracking fluorescence microscopy in mitochondria reveals highly dynamic but confined movement of Tom40. *Scientific reports* **1**.

[82] **Chang CR, Blackstone C**. 2007. Cyclic AMP-dependent Protein Kinase Phosphorylation of Drp1 Regulates Its GTPase Activity and Mitochondrial Morphology. *J Biol Chem* **282**: 21583–21587.

[83] **Han XJ, Lu YF, Li SA, Kaitsuka T,** et al. 2008. CaM kinase Iα–induced phosphorylation of Drp1 regulates mitochondrial morphology. *J Cell Biol* **182**: 573–585.

[84] **Wang W, Wang Y, Long J, Wang J,** et al. 2012. Mitochondrial fission triggered by hyperglycemia is mediated by ROCK1 activation in podocytes and endothelial cells. *Cell Metab* **15**: 186–200.

[85] **Taguchi N, Ishihara N, Jofuku A, Oka T,** et al. 2007. Mitotic Phosphorylation of Dynamin-related GTPase Drp1 Participates in Mitochondrial Fission. *J Biol Chem* **282**: 11521–11529.

[86] **Cho B, Cho HM, Kim HJ, Jeong J,** et al. 2014. CDK5-dependent inhibitory phosphorylation of Drp1 during neuronal maturation. *Exp Mol Med* **46**: e105.

[87] **Qi X, Disatnik MH, Shen N, Sobel RA,** et al. 2011. Aberrant mitochondrial fission in neurons induced by protein kinase Cδ under oxidative stress conditions in vivo. *Mol Biol Cell* **22**: 256–265.

[88] **Yu T, Jhun BS, Yoon Y**. 2011. High-glucose stimulation increases reactive oxygen species production through the calcium and mitogen-activated protein kinase-mediated activation of mitochondrial fission. *Antioxidants & redox signaling* **14**: 425–437.

[89] **Cereghetti GM, Stangherlin A, de Brito OM, Chang CR,** et al. 2008. Dephosphorylation by calcineurin regulates translocation of Drp1 to mitochondria. *Proc Natl Acad Sci USA* **105**: 15803–15808.

[90] **Braschi E, Zunino R, McBride HM**. 2009. MAPL is a new mitochondrial SUMO E3 ligase that regulates mitochondrial fission. *EMBO Rep* **10**: 748–754.

[91] **Figueroa-Romero C, Iiguez-Lluh JA, Stadler J, Chang CR,** et al. 2009. SUMOylation of the mitochondrial fission protein Drp1 occurs at multiple nonconsensus sites within the B domain and is linked to its activity cycle. *FASEB J* **23**: 3917–3927.

[92] **Gawlowski T, Suarez J, Scott B, Torres-Gonzalez M,** et al. 2012. Modulation of Dynamin-related Protein 1 (DRP1) Function by Increased O-linked--N-acetylglucosamine Modification (O-GlcNAc) in Cardiac Myocytes. *J Biol Chem* **287**: 30024–30034.

[93] **Wasiak S, Zunino R, McBride HM**. 2007. Bax/Bak promote sumoylation of DRP1 and its stable association with mitochondria during apoptotic cell death. *J Cell Biol* **177**: 439–450.

[94] **Yonashiro R, Ishido S, Kyo S, Fukuda T,** et al. 2006. A novel mitochondrial ubiquitin ligase plays a critical role in mitochondrial dynamics. *EMBO J* **25**: 3618–3626.

[95] **Nagashima S, Tokuyama T, Yonashiro R, Inatome R,** et al. 2014. Roles of mitochondrial ubiquitin ligase MITOL/MARCH5 in mitochondrial dynamics and diseases. *J Biochem* .

[96] **Gottlieb E**. 2006. OPA1 and PARL Keep a Lid on Apoptosis. *Cell* **126**: 27–29.

[97] **Santo-Domingo J, Giacomello M, Poburko D, Scorrano L,** et al. 2013. OPA1 promotes pH flashes that spread between contiguous mitochondria without matrix protein exchange. *EMBO J* **32**: 1927–1940.

[98] **Lee H, Yoon Y**. 2014. Transient Contraction of Mitochondria Induces Depolarization through the Inner Membrane Dynamin OPA1. *J Biol Chem* .

[99] **de Brito OM, Scorrano L**. 2008. Mitofusin 2 tethers endoplasmic reticulum to mitochondria. *Nature* **456**: 605–610.

[100] **Pich S, Bach D, Briones P, Liesa M,** et al. 2005. The CharcotMarieTooth type 2A gene product, Mfn2, up-regulates fuel oxidation through expression of OXPHOS system. *Hum Mol Genet* **14**: 1405–1415.

[101] **Jahani-Asl A, Cheung ECC, Neuspiel M, MacLaurin JG,** et al. 2007. Mitofusin 2 Protects Cerebellar Granule Neurons against Injury-induced Cell Death. *J Biol Chem* **282**: 23788–23798.

[102] **Ryu SW, Choi K, Park JH, Park YM,** et al. 2012. Mitofusin 1 inhibits an apoptosis-associated amino-terminal conformational change in Bax, but not its mitochondrial translocation, in a GTPase-dependent manner. *Cancer Lett* **323**: 62 – 68.

[103] **Frank S, Gaume B, Bergmann-Leitner ES, Leitner WW,** et al. 2001. The role of dynamin-related protein 1, a mediator of mitochondrial fission, in apoptosis. *Dev Cell* **1**: 515–525.
